# Sequence of the *Gonium pectorale* Mating Locus Reveals a Complex and Dynamic History of Changes in Volvocine Algal Mating Haplotypes

**DOI:** 10.1534/g3.115.026229

**Published:** 2016-02-22

**Authors:** Takashi Hamaji, Yuko Mogi, Patrick J. Ferris, Toshiyuki Mori, Shinya Miyagishima, Yukihiro Kabeya, Yoshiki Nishimura, Atsushi Toyoda, Hideki Noguchi, Asao Fujiyama, Bradley J. S. C. Olson, Tara N. Marriage, Ichiro Nishii, James G. Umen, Hisayoshi Nozaki

**Affiliations:** * Donald Danforth Plant Science Center, St Louis, Missouri 63132; † Department of Biological Sciences, Graduate School of Science, University of Tokyo 113-0033, Japan; ‡ Department of Ecology and Evolutionary Biology, University of Arizona, Tucson, Arizona 85721; § Symbiosis and Cell Evolution Laboratory and Comparative Genomics Laboratory, National Institute of Genetics, Mishima, Shizuoka 411-8540, Japan; †† Department of Botany, Graduate School of Science, Kyoto University, Kyoto 606-8502, Japan; ‡‡ Division of Biology, Kansas State University, Manhattan, Kansas 66506; §§ Department of Chemistry, Biology, and Environmental Sciences, Faculty of Science, Nara Women’s University, Nara 630-8506, Japan

**Keywords:** *Gonium*, *Chlamydomonas*, *Volvox*, mating locus, evolution, Genetics of Sex

## Abstract

Sex-determining regions (SDRs) or mating-type (*MT*) loci in two sequenced volvocine algal species, *Chlamydomonas reinhardtii* and *Volvox carteri*, exhibit major differences in size, structure, gene content, and gametolog differentiation. Understanding the origin of these differences requires investigation of *MT* loci from related species. Here, we determined the sequences of the *minus* and *plus MT* haplotypes of the isogamous 16-celled volvocine alga, *Gonium pectorale*, which is more closely related to the multicellular *V. carteri* than to *C. reinhardtii*. Compared to *C. reinhardtii MT*, *G. pectorale MT* is moderately larger in size, and has a less complex structure, with only two major syntenic blocs of collinear gametologs. However, the gametolog content of *G. pectorale MT* has more overlap with that of *V. carteri MT* than with *C. reinhardtii MT*, while the allelic divergence between gametologs in *G. pectorale* is even lower than that in *C. reinhardtii*. Three key sex-related genes are conserved in *G. pectorale MT*: *GpMID* and *GpMTD1* in *MT*–, and *GpFUS1* in *MT*+. GpFUS1 protein exhibited specific localization at the *plus*-gametic mating structure, indicating a conserved function in fertilization. Our results suggest that the *G. pectorale–V. carteri* common ancestral *MT* experienced at least one major reformation after the split from *C. reinhardtii*, and that the *V. carteri* ancestral *MT* underwent a subsequent expansion and loss of recombination after the divergence from *G. pectorale*. These data begin to polarize important changes that occurred in volvocine *MT* loci, and highlight the potential for discontinuous and dynamic evolution in SDRs.

Sex determining regions (SDRs) can exhibit complex patterns of molecular evolution that are distinct from those of autosomes (Bachtrog *et al.* 2011). The origins of SDR architecture and function have been studied in diverse species, but our understanding of the evolutionary processes that shape SDRs is still limited. Recombination suppression—a common property of both haploid and diploid SDRs—reinforces linkage between sex-related genes within the SDR, and promotes accumulation of sexually antagonistic alleles linked to the SDR (Bachtrog *et al.* 2011; Immler and Otto 2015). SDRs often contain genomic rearrangements that may be either the cause, or consequence, of recombination suppression between heteromorphic haplotypes or sex chromosomes. The deleterious effects of suppressed recombination are also seen in SDRs where Y or Z chromosomes in diploid mating systems may undergo degeneration (Charlesworth *et al.* 2005). Much less is known about the long-term evolutionary histories of SDRs in haploid mating systems where SDRs also undergo degeneration, but where limited data are available (Yamato *et al.* 2007; Bachtrog *et al.* 2011; Sun *et al.* 2012; McDaniel *et al.* 2013; Ahmed *et al.* 2014).

Volvocine algae offer unique advantages as a model for the evolution of SDRs. Like many protists, volvocine algae are haploid and capable of both asexual (vegetative) and sexual reproduction. Environmental or hormonal cues trigger gametic differentiation, which in heterothallic species is controlled by a heteromorphic mating-type (*MT*) locus with two haplotypes: *plus*/*minus*, or male/female (see below). Volvocine algae also encompass a range of morphologies from unicellular genera such as *Chlamydomonas*, to *Volvox*, which have thousands of cells, and exhibit germ–soma differentiation and other developmental innovations that result in a functionally integrated multicellular colony (summarized in Figure 1). Sexual reproduction coevolved with colony size in volvocine algae, whose mating strategies include isogamy in unicellular and small colonial genera (*Chlamydomonas*, *Gonium*, and *Yamagishiella*), anisogamy in intermediate-sized genera (*Eudorina* and *Pleodorina*), and oogamy in the largest, and most differentiated, genus *Volvox*. Transitions between heterothallism (genetic sex determination) and homothallism (self-mating) have also occurred within volvocine algae (Coleman 2012) (Figure 1). The well-established phylogenetic relationships between volvocine species enable specific traits and innovations to be ordered and mapped onto a coherent evolutionary framework (Nozaki *et al.* 2000; Herron and Michod 2008).

**Figure 1:**
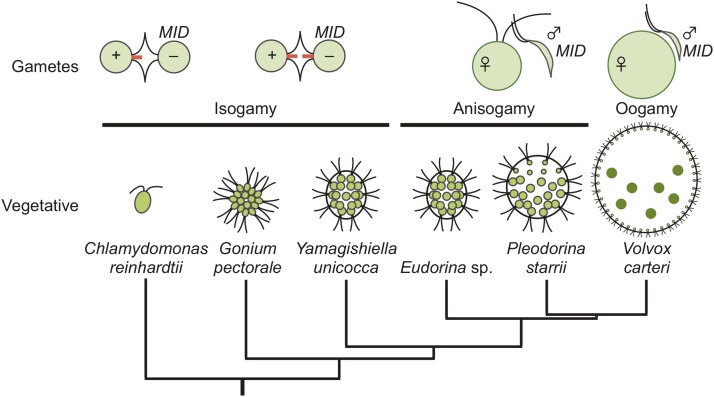
A schematic diagram for phylogenetic relationships of selected volvocine species based on Nozaki *et al.* (2000) and Herron and Michod (2008). The top row illustrates gamete type and structure. Tubular mating structures in isogamous gametes are indicated with red bars at the flagellar base. The possession of a *MID* gene is shown next to the *minus* mating type or male gametes. The lower row of cartoons depicts vegetative morphology (not to scale) for the indicated species.

The full genome sequences, including regions of both haplotypes of *MT*, were previously described for two heterothallic species: isogamous unicellular *C*. *reinhardtii*, and oogamous multicellular *Volvox carteri* (Figure 2) (Merchant *et al.* 2007; Ferris *et al.* 2010; Prochnik *et al.* 2010). Importantly, the genetic and molecular bases of *MT* differentiation in volvocine algae, including functions of mating locus genes, have been investigated (Ferris and Goodenough 1994, 1997; Ferris *et al.* 1996, 2002; De Hoff *et al.* 2013; Geng *et al.* 2014). *MT* haplotypes (*plus/minus* or male/female) segregate as single Mendelian traits, but the loci themselves are multigenic regions in which recombination is suppressed. The core of the mating locus is the rearranged (R) domain, where gene order and arrangements between the two haplotypes are noncolinear (Umen 2011). In *C*. *reinhardtii*, the *plus* and *minus* haplotypes of *MT* reside on chromosome 6 (Ferris and Goodenough 1994; Ferris *et al.* 2002; De Hoff *et al.* 2013), while *V. carteri MT* is located in a region of linkage group I that shares a common origin with *C. reinhardtii* chromosome 6 (Ferris *et al.* 2010). Despite their shared chromosomal location, *V*. *carteri MT* is much larger in size than *C*. *reinhardtii MT*, and shows a far higher degree of differentiation between gametologs (allelic gene pairs residing in *MT*) (Ferris *et al.* 2010). This discrepancy raises many questions about why the two *MT* loci differ so much in genetic divergence and recombination potential (Charlesworth and Charlesworth 2010; Umen 2011). One important feature of *C. reinhardtii MT* that is not found in *V. carteri MT* is low-frequency gene conversion between gametologs that acts to reduce allelic differentiation (De Hoff *et al.* 2013).

**Figure 2:**
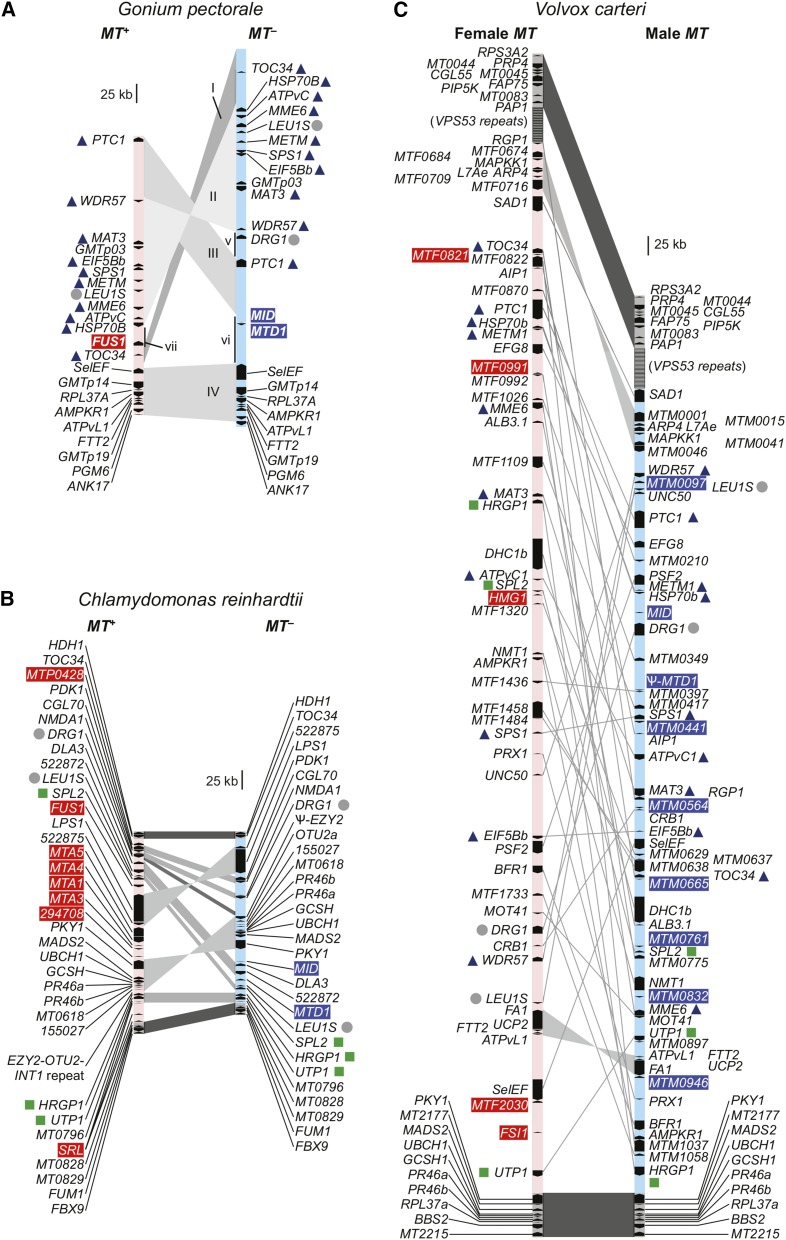
Schematic of volvocine mating loci for (A) *Gonium pectorale*, (B) *Chlamydomonas reinhardtii* (modified from De Hoff *et al.* 2013), and (C) *Volvox carteri* (modified from Ferris *et al.* 2010), with rearranged (R) domains in light blue (*minus*/male) or pink (*plus*/female). For *G. pectorale MT*, syntenic blocs are indicated by gray shading, and labeled with upper case roman numbers (I–IV), while sequences that are unique to one of the two mating haplotypes are indicated with lower case roman numbers (v–vii). Red and blue shading on gene names indicates *plus*/female and *minus*/male specific genes, respectively. Gray dots beside gene names indicate those found in the R domains of all three species; dark blue triangles indicate presence of gene in R domain of *G. pectorale* and *V. carteri* only; green squares indicate presence of gene in R domain of *C. reinhardtii* and *V. carteri* only. No R domain genes are shared exclusively by *G*. *pectorale* and *C*. *reinhardtii*.

The *C. reinhardtii MID* (*minus*-dominance) gene is present only in the *MT*– haplotype, and plays a critical role in determining mating type (Ferris and Goodenough 1997). Recently, the *V. carteri MID* gene which is found only in the male *MT* haplotype (Ferris *et al.* 2010) was also shown to govern critical aspects of sex determination (Geng *et al.* 2014). Moreover, *MID* genes, have been found in the *minus* or male mating haplotypes of other heterothallic volvocine algae including several *Gonium* species (Hamaji *et al.* 2008, 2013a; Setohigashi *et al.* 2011), and *Pleodorina starrii* (Nozaki *et al.* 2006), suggesting that the genetic basis of sex- or mating-type determination is conserved throughout the volvocine lineage (Figure 1). Other than the *MID* gene, no sex-related genes are conserved between the *MT* loci of *V. carteri* and *C. reinhardtii*. The strikingly different sizes, gametolog contents, gametolog differentiation, and recombination behaviors of *C. reinhardtii vs. V. carteri MT* loci raise questions about how these two SDRs, which appear to have arisen from a common ancestral SDR region, diverged so markedly from each other. To answer these questions requires information on volvocine algal *MT* loci from additional species in order to begin reconstructing ancestral states and polarizing changes within the lineage.

*Gonium pectorale* is a small isogamous colonial volvocine species that is more closely related to *V. carteri* than it is to *C. reinhardtii* (Nozaki *et al.* 2000). As such, it may represent an informative taxon for reconstructing the evolution of *MT* loci in the volvocine lineage. Previous work on *G. pectorale* identified *MT*– homologs of *MID* and of *MTD1*, genes that are both also found in the *MT*– haplotype of *C. reinhardtii* (Hamaji *et al.* 2008, 2009). To date, no homologs of *MT+* limited genes from *C. reinhardtii* have been found in other volvocine species, including the gene *FUS1*, which encodes a membrane-bound protein that localizes to the mating apparatus or “fertilization tubule,” and is required for fusion of *plus* and *minus* gametes (Ferris *et al.* 1996, 1997; Misamore *et al.* 2003).

Here we report the sequence of *G*. *pectorale MT*+ and *MT*– haplotypes derived from a combination of chromosomal walking, and whole genome sequencing. The overall structure of *G. pectorale MT* was found to differ from *C*. *reinhardtii MT* and *V*. *carteri MT* in terms of structural rearrangements between haplotypes. It has fewer rearranged sequence blocs than *C. reinhardtii MT*, and no large autosomal insertions in either haplotype. *G. pectorale* shares, in common with *C. reinhardtii*, the presence of sex-limited genes *MID* and *MTD1* in its *MT*- haplotype (Hamaji *et al.* 2008, 2009), and a *FUS1* homolog in its *MT+* haplotype, whose gene product, GpFUS1, localizes to the fertilization tubule, indicating homologous function with CrFUS1. As in *C. reinhardtii*, gametolog differentiation in *G. pectorale MT* is minimal. However, the gametolog gene complement of *G. pectorale MT* is more similar to that of *V. carteri MT* than that of *C. reinhardtii MT*. Taken together, these data enable us to reconstruct a minimal set of changes in the *MT* region that led to their current states. Our findings suggest a dynamic, and most likely punctuated, history of volvocine *MT* evolution that involves episodes of structural reconfiguration in which gametolog content and syntenic blocs change, but where mating-related genes remain linked to their ancestral *MT* haplotypes. The expansion, gametolog differentiation, and loss of *MTD1* and *FUS1* genes that characterize *V. carteri MT* likely occurred after the *G. pectorale* and *V. carteri* lineages split.

## Materials and Methods

### Strains used

The genomic DNA sequence data presented here come from previously described strains of *G*. *pectorale*: Kaneko3 (*minus*) and Kaneko4 (*plus*) (Yamada *et al.* 2006; Hamaji *et al.* 2008, 2013b), K3-F3-4 (*minus*), and K4-F3-4 (*plus*), which are F3 hybrid progeny from Kaneko3 and Kaneko4 as described previously (Hamaji *et al.* 2013b).

### BAC library construction

DNA plugs were prepared from both mating types (Kaneko4: *plus*; Kaneko3: *minus*) in 1% SeaPlaque GTG agarose (Cambrex Bio Science Rockland), treated with Pronase E (Sigma), and washed thoroughly as described (Ferris *et al.* 2010). BAC library production was performed at the Clemson University Genome Institute. The DNA plugs were partially digested with *Eco*RI, and then DNA fragments size-fractioned (to ca. 120 kb) by pulse-field gel electrophoresis were ligated into pIndigoBAC536. Single colonies of *Escherichia coli* strain DH10b transformed with *G. pectorale* genomic fragment-containing BACs were picked, spotted on nylon membranes (or “BAC filters”), and stored frozen in glycerol at –80°C (total BAC number, *plus*: 27,648; *minu*s: 18,432, estimated 24× and 16× coverage of *G. pectorale plus* and *minus* genome, respectively).

### BAC screening and chromosomal walking

BAC filters were hybridized with probes labeled and detected with CDP-Star (GE Healthcare) or DIG-labeling kit (Roche). Positive signals were confirmed by direct PCR with probe-specific primers on the individual clones. BACs of true positives were column-purified from 5 ml cultures, and end-sequenced with M13 Forward and Reverse primers. Each end sequence is designed for a pair of specific primers, which was used to determine the relative locations of overlapping BACs. Primer pairs from both distal ends were selected to label probes for the next round of chromosomal walking. Inverse PCR (Sambrook and Russell 2001) and TAIL-PCR (Liu and Whittier 1995) were performed to identify flanking sequences when no additional BAC clones could be identified. *GpMTD1* probes were the same as those used for the *GpMTD1* DNA gel-blot analysis (Hamaji *et al.* 2009). The PCR product of GPLEUFATG and GPLEURTAA was labeled to screen for *LEU1S* alleles (Supplemental Material, Table S1). Two gametologs (*WDR57* and *DRG1*) and their flanking regions, which were not obtained in the *MT*+ BAC assembly, were obtained by genomic PCR using specific primers based on the *MT*– alleles. The linkage of *MT*+ *DRG1* was determined by recombination scores as described below. Abundant repetitive regions flanking the *MT* assembly prevented further chromosomal walking to connect directly to autosomal sequences.

### Shotgun sequencing and assembly

BAC DNA was mechanically sheared either with nebulizers (for 30 sec) in the TOPO shotgun sub-cloning kit (Invitrogen), or with sonication. Blunt-end fragments were subcloned into pCR4, pCRII (Invitrogen), or pUC118 (Takara Bio). Shearing with sonication, and pUC118 subcloning, were performed by the Kazusa DNA Research Institute. Shotgun subclones were sequenced from both ends by the Research Resource Center (BSI, RIKEN), or the Kazusa DNA Research Institute. Raw sequence data were base-called, vector-trimmed for each, end-clipped to remove low-quality regions, and assembled by CodonCode Aligner (CodonCode: http://www.codoncode.com/aligner/). Assembled contigs were queried against *C*. *reinhardtii* V4 protein models, or *V*. *carteri* protein models (Ver. 2, JGI and male and female *MT*, NCBI) using BLASTX to identify protein coding genes (Merchant *et al.* 2007; Ferris *et al.* 2010).

### Annotation

Gene models were generated using Fgenesh (Salamov and Solovyev 2000) (http://linux1.softberry.com/berry.phtml) with the “Chlamydomonas” option, and then manually edited based on similarity to *C. reinhardtii* (JGI V4 protein models: http://genome.jgi-psf.org/Chlre4/Chlre4.home.html) or *V. carteri* (male and female *MT* genes available in GenBank: http://www.ncbi.nlm.nih.gov/genbank/) homologs.

### GpFUS1 identification and characterization

BLASTX search of the *G. pectorale MT* locus contigs using *C. reinhardtii* proteins (ver. 4) identified a *FUS1*-like sequence in *MT*+. RT-PCR fragments of 5′-/3′-RACE were cloned into TOPO TA vector to determine the full-length cDNA sequence. Subsequently, genomic sequences were obtained from *plus* BAC 71M20. The *G. pectorale FUS1* full-length cDNA sequence was determined by the same method as *GpMTD1* (specific primers used: GpFUS1AF; GpFUS1AR; GpFUS1BF; GpFUS1BR; GpFUS1CF; GpFUS1CR). The initially gapped genomic sequence of *GpFUS1* was filled by genomic PCR. Hydrophobicity with window size of nine, and theoretical protein isoelectric point (pI)/molecular weight (MW) were calculated with ProtScale and Compute pI/Mw in ExPASY (Gasteiger *et al.* 2003) (http://au.expasy.org/).

### Anti-GpFUS1 antibody production, Western blotting, and immunofluorescence

The coding region corresponding to amino acid residues 90–489 out of the predicted 824-aa sequence of GpFUS1 protein was amplified by PCR with primers YM001F and YM001R. The resultant DNA was cloned into pET100/D-TOPO vector (Invitrogen), and expressed as a His-tag fusion protein. The recombinant protein was isolated with a HisTrap HP column (GE Healthcare). Purified protein was injected into two rabbits for antibody production (Riken, Saitama, Japan). The rabbit antisera were affinity-purified with a 6His-GpFUS1-coupled HiTrap NHS-activated HP column (GE Healthcare), according to the manufacturer’s instructions. The final concentration of antibody was ≫0.25 mg/ml.

To stain GpFUS1 protein expressed in gametes, indirect immunofluorescence microscopy assay and Western blot analyses were performed as previously described after dibutyryl-cyclic AMP (db-cAMP) treatment (Mogi *et al.* 2012).

### Linkage mapping

DNA samples of 78 F1 progeny (haploid recombinants from F1 meiosis) derived from *G*. *pectorale* Mongolia 1×4 strains (described previously) (Hamaji *et al.* 2008) were genotyped for markers designed based on polymorphic genome sequences. Primers, restriction enzymes, and accession numbers in PCR-RFLP analyses are listed in Table S1. The PCR conditions were 2 min 94°, 35 cycles of 30 sec at 94°, 30 sec at 53° (for *DRG1*, 50°), and 30 sec at 72°, followed by 7 min at 72° with the same reaction composition as reported previously (Ferris *et al.* 2010). Restriction digests of the PCR products were performed (Sambrook and Russell 2001) to identify the two genotypes.

### Molecular evolutionary analyses

Previously reported genes linked to, or residing in, *MT* loci of *C*. *reinhardtii* or *V*. *carteri* were queried by TBLASTN searching (Altschul *et al.* 1990) of the *G*. *pectorale de novo* draft genome assembly (Hanschen *et al.* 2016). Genome-wide synteny comparison between *C*. *reinhardtii* (v4 assembly) and *G*. *pectorale* was done using SyMAP (Soderlund *et al.* 2006, 2011).

*MT* shared genes (*i.e.*, gametologs) were analyzed as previously described (Ferris *et al.* 2010). Coding or genomic sequences were aligned using ClustalX 2.0 (Larkin *et al.* 2007) and alignments adjusted manually. Divergence scores were calculated using yn00 from the PAML4 package (Yang 2007); nonsynonymous and synonymous site divergence (d_N_ and d_S_, respectively) of aligned coding sequences of gametologs was estimated according to Yang and Nielsen (2000) with equal weighting between pathways, and the same codon frequency for all pairs. Sliding window analysis of each gametolog was performed using DnaSP v5.10.1 (Librado and Rozas 2009) for Pi (Nei 1987) over the aligned genomic sequences from both haplotypes from start to stop codons in 100-base windows with a 25-base interval.

### Data availability

DDBJ/ENA/GenBank accessions: LC062386 (*G*. *pectorale DRG1 plus* haplotype); LC062718 (*MT*- scaffold); LC062719 (*MT*+ scaffold).

## Results and Discussion

### Size, structure, and gene content of G. pectorale MT

BAC contigs containing *G*. *pectorale MT* sequences comprised approximately 500 kb for *MT*– and 360 kb for *MT*+ (Figure 2A, Figure S1, and Table 1). Although these contigs could not be directly connected to autosomal scaffolds, based on their genetic contents and other criteria outlined below, it is likely they contain all or most of the rearranged (R) domain of *G. pectorale MT*. Contigs from both haplotypes were sequenced and used for BLASTX and TBLASTX queries against the *C. reinhardtii* proteome, and against the *V. carteri MT* locus DNA sequence, respectively. These searches detected 24 *G. pectorale MT* genes, 21 of which are gametolog pairs, meaning that an allele is present in both mating haplotypes. Three additional mating-type-specific genes, *MID*, *MTD1*, and *FUS1* were also present, with *MID* and *MTD1* in *MT*–, and *FUS1* in *MT+* (Figure 2A and Table S2). The gene contents of *G. pectorale MT* are described in more detail below.

**Table 1:** Genome and mating locus properties of *Gonium pectorale*, *Chlamydomonas reinhardtii*, and *Volvox carteri*

	*Gonium pectorale*			*Chlamydomonas reinhardtii*			*Volvox carteri*		
	*MT*+	*MT*-	Whole	*MT*+	*MT*–	Whole	Female	Male	Whole
Size (Mb)	0.366	0.499	148.8	0.310	0.204	111.1	1.51	1.13	131.1
%GC	59.7	61.0	64.5	60	61	64.1	52	53	56.1
Gene density [genes/Mb]	57.38	46.09	120.9	74.25	117.6	159.6	39	54	114.1
Introns / gene	9.62	9.09	6.50	7.1	6.37	7.46	9	8	6.29
Average intron length (bp)	175.8	278.50	349.83	354.17	358.70	420.01	618	584	399.50

Three rearranged sequence blocs (designated I–III in Figure 2A and Figure S2) define its R domain of *G*. *pectorale MT* with the largest, bloc II, being a > 200-kb inversion that retains collinearity of its 10 resident gametologs, and whose intergenic regions contain a few interspersed repeats (Figure 2A and Figure S2). Bloc III contains a single gene, *PTC1* which is flanked by extensive noncoding repeats that comprise the majority of this subregion. While the coding regions of *PTC1* are nearly identical between gametologs, the 3′-UTR of the *MT*– allele is extended compared to the *MT+* allele, leading to a larger predicted gene size (Figure 2A). Bloc I contains the *TOC34* gene, whose gametolog pairs have low similarity between gametologs compared with bloc II gametologs (Figure 2A; discussed below), and has almost no similarity in intergenic regions (Figure 2A and Figure S2). Bloc IV (Figure 2A and Figure S2) is almost completely collinear between haplotypes, and, as described below, likely represents the centromere-proximal autosomal flank of *MT*. The only significant difference between the two mating haplotypes in bloc IV is at its proximal end next to the R domain, where a large intron is present in the *MT*– allele of *SelEF* at its 3′ end, but absent from the *MT*+ allele, accounting for their different sizes (Figure 2A and Figure S2). The distal-most gene from the R domain in bloc IV, *ANK17*, was incompletely assembled in each haplotype, with differences in the length of assembled sequences accounting for the size difference between its two alleles (Figure 2A). On the other side of *MT* (top end in Figure 2A), no additional sequences distal to *TOC34* in *MT*– and *PTC1* in *MT+* were found, and it is likely that, beyond this region are repeats and/or telomeric sequences, as is the case in *V. carteri* (Ferris *et al.* 2010). Interspersed between the syntenic blocs I–IV are three regions (v–vii) of *G*. *pectorale MT* that are unique to one of the two haplotypes. Region vi of *MT*– contains homologs of two *C. reinhardtii* sex-determining genes, *GpMTD1* and *GpMID*, which have been previously described (Hamaji *et al.* 2008, 2009). Region vii of *MT+* contains a homolog of *FUS1*, a *C*. *reinhardtii* fertilization protein. Characterization of *GpFUS1* is presented below. Region v initially appeared to be unique to *MT*– and contains a single gene, *DRG1*, whose orthologs in *C. reinhardtii* and *V. carteri* are present as gametolog pairs in their respective *MT* loci (Ferris *et al.* 2002, 2010; De Hoff *et al.* 2013) but which have no known function in the sexual cycle. A *MT*+ linked allele of *DRG1* was subsequently identified, as described below.

Overall, the haplotype bloc structure of *G. pectorale MT* is notable for being completely different from that of *C. reinhardtii* or *V. carteri MT*, with fewer overall rearrangements than in *C. reinhardtii* (Figure 2) (Ferris *et al.* 2010; De Hoff *et al.* 2013). Also, apparently absent from *G. pectorale MT* are the large autosomal insertions and tandem repeats that are found in *C. reinhardtii MT* (De Hoff *et al.* 2013). Although it is larger than *C. reinhardtii MT*, the structure of *G. pectorale MT* is simpler, with fewer sequence rearrangements and insertions, consistent with a younger age. It is also very different from *V. carteri MT*, which is characterized by much more extensive sequence rearrangements, and few collinear blocs of gametologs. Taken together, our findings suggest a nonlinear trajectory of *MT* evolution in volvocine algae, as discussed further below.

### Genomic location and completeness of the G. pectorale MT assembly by linkage mapping

Although we could not connect the *G. pectorale MT* assembly physically to other scaffolds in the *G. pectorale* whole genome assembly, we identified a linked 2.8 Mb autosomal scaffold (scaffold00010) that is syntenic to a portion of *C*. *reinhardtii MT*-containing chromosome 6 (Table 2 and Figure S3). Recombination was measured between each terminus of *G. pectorale* scaffold00010 and *MT*, and revealed that one of the termini did not recombine *MT* (0/78) but that the other one did, with a genetic distance of 17 cM (13/78 recombinants). Based on physical estimates from two independent mapping intervals (Figure S3 and Table 2), the proximal end of scaffold00010 is less than 100 kb from *MT*. Ferris *et al.* (2010) reported a common chromosomal origin for regions containing *MT* loci of *C*. *reinhardtii* (Chr. 6), and *V*. *carteri* (Chr. 1). Our findings here further support this idea and show that, despite large changes in structure and gene content, *MT* loci appear to have remained stably associated with a single chromosomal location throughout the volvocine lineage.

**Table 2:** Linkage mapping of progeny from *Gonium pectorale* Mongolia 1 and 4

Character*^b^*	*RABB1*	*MTF1109*	*PRX1*	*UNC50*	*IDA5*	*PR46a*	*ALB3.1*	*LEU1S*	*MID*	*MT*	*PGM6*	*SpoVS*	*PLC*
Scaffold*^a^*	5	3	1	2	97	7	7	MT	MT	Phenotype*^d^*	MT	10	10
Cre. Chr.*^c^*	2	6	6	6	13	6	6	6	6	—	6	6	6
*RABB1*		34/78	42/78	38/77	42/78	37/77	38/78	40/78	40/78	35/69	40/78	40/78	35/78
*MTF1109*			34/78	43/77	36/78	33/77	34/78	44/78	44/78	40/69	44/78	44/78	49/78
*PRX1*				34/77	40/78	32/77	26/78	38/78	38/78	36/69	38/78	38/78	41/78
*UNC50*					42/77	33/76	37/77	38/77	38/77	33/68	38/77	38/77	39/77
*IDA5*						39/77	36/78	48/78	48/78	42/69	48/78	48/78	49/78
*PR46a*							12/77	44/77	44/77	39/68	44/77	44/77	45/77
*ALB3.1*								40/78	40/78	38/69	40/78	40/78	41/78
*LEU1S*									0/78	0/69	0/78	0/78	13/78
*MID*										0/69	0/78	0/78	13/78
*MT*											0/69	0/69	12/69
*PGM6*												0/78	13/78
*PLC*													13/78

Crossing revised from a former experiment (Hamaji *et al.* 2008). Values given as ratio of recombinant progeny to total progeny. MT, mating type.

a Scaffold in *Gonium pectorale* genome assembly.

b Genotypic or phenotypic characters assayed.

c Physical positions in *Chlamydomonas reinhardtii* chromosome for homologs of genetic markers.

d Mating phenotype.

The near completeness of the *G. pectorale* R domain assembly was supported by our finding gametolog partners in each mating haplotype for every gene other than the three genes described above (*MID*, *MTD1*, and *FUS1*), which are already known to be sex-limited homologs in *C. reinhardtii* and/or *V. carteri*. With the exception of *DRG1*, both gametolog copies are in *MT* scaffolds. In addition, when we searched for *G. pectorale* homologs of genes present in, and around, *MT* from *C. reinhardtii* and *V. carteri*, we were able to locate nearly all of them in *G. pectorale* autosomal scaffolds, or within its *MT* assembly. Although we could not find a copy of *DRG1* in either the *MT*+ genome scaffolds or in the *G. pectorale* whole genome assembly (Hanschen *et al.* 2016), we reasoned that a *MT+* allele might be in a region of the *MT+* haplotype that was not assembled. Using PCR with primers designed to amplify the *MT*– copy of *GpDRG1*, we identified a *MT+ DRG1* allele (see *Materials and Methods*), and found that it is tightly linked to *MT* (0/78 recombinants). Besides *DRG1*, there are no other “orphan” gametologs in our *MT* assembly, and no additional sex-limited genes other than *GpMID*, *GpMTD1*, and *GpFUS1*. Taken together, our data suggest that the *MT* assembly shown in Figure 2 is nearly complete, as is the inventory of *G. pectorale MT*-linked genes (Table S3).

### Evolution of gene contents and structure of volvocine MT loci

*G. pectorale* homologs of many *V. carteri* and *C. reinhardtii* mating type genes (*i.e.*, gametologs) are autosomal, meaning that, in *G. pectorale*, these genes reside outside of the *MT* locus (Table S3). Overall, the gametolog contents of *G. pectorale MT* show much more overlap with *V. carteri* gametologs (19 out of 20 in common) than with *C. reinhardtii* gametologs (two out of 20 in common) showing that *G. pectorale MT* and *V*. *carteri MT* diverged more recently (Figure 1). Besides the sex-limited genes already mentioned above (*MTD1*, *MID*, and *FUS1* in *C. reinhardtii*; *MID* in *V. carteri*), *C. reinhardtii* and *V. carteri* both have additional sex-limited genes that we searched for in *G. pectorale* (Table S2). Homologs of *EZY2* or *MTA1* from *C. reinhardtii* (Ferris and Goodenough 1994; Ferris *et al.* 2002), or *FSI1* and *HMG1* from *V. carteri* (Ferris *et al.* 2010), could not identified anywhere in the *G*. *pectorale* whole genome sequence or *MT* region. However, a *G. pectorale* homolog of the *C*. *reinhardtii MT+* gene *OTU2* (De Hoff *et al.* 2013) was found on an autosomal scaffold (scaffold00113). In summary, compared to either *C. reinhardtii* or *V. carteri*, *G. pectorale MT* is the most minimal in terms of structural rearrangements, and in its sex-limited gene content.

### Molecular evolution of G. pectorale MT genes

**Figure 3:**
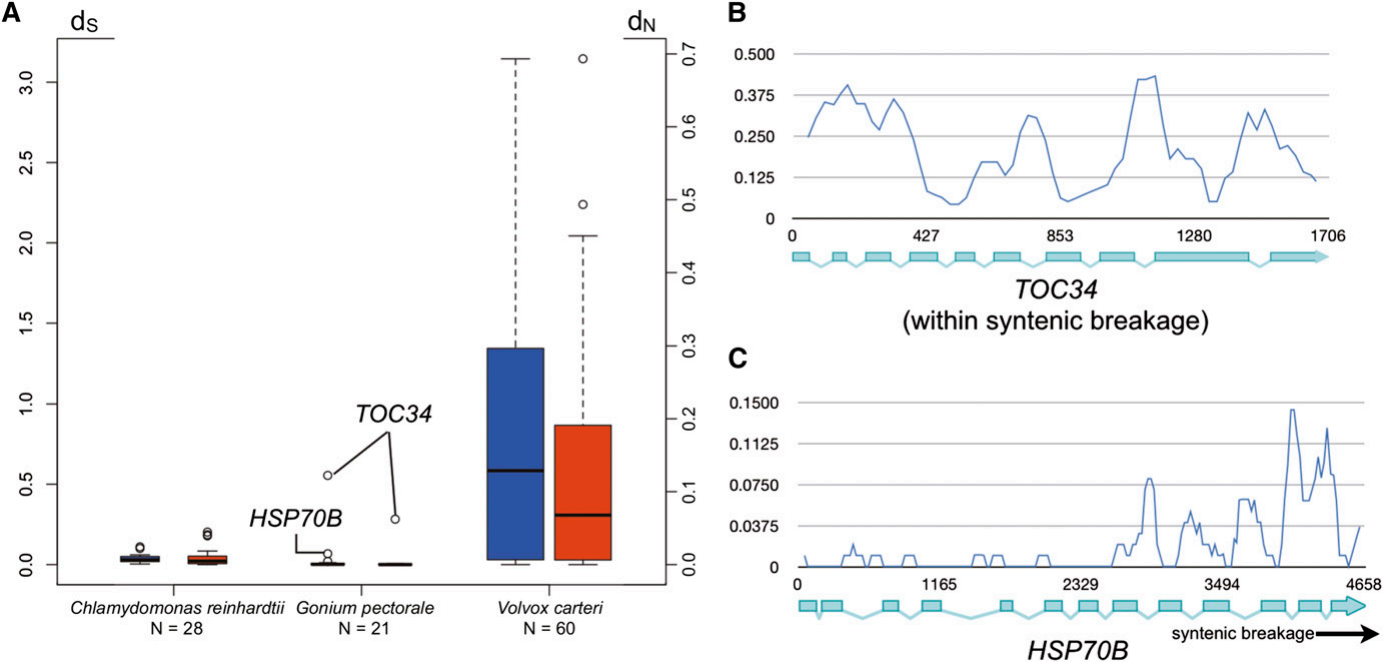
Molecular evolutionary analyses of volvocine algal gametologs. (A) Box-whisker plots comparing the distributions of d_S_ (blue) and d_N_ (red) values for *Chlamydomonas reinhardtii*, *Gonium pectorale*, and *Volvox carteri*. Open dots in *G. pectorale* are values for indicated genes. (B) and (C) Sliding window plots of gametolog similarity (Pi) for *TOC34* (B) or *HSP70B* (C). Position within each gene is indicated on *x*-axis by bp number beginning with the start codon.

Previous work on *C. reinhardtii* and *V. carteri MT* loci revealed very different divergences between gametolog pairs, with high divergences for most *V. carteri* pairs, and low divergences for *C. reinhardtii* pairs (Ferris *et al.* 2010). It was subsequently found that low frequency gene conversion between gametolog sequences plays a role in maintaining sequence homogeneity between the nominally nonrecombining regions of *C. reinhardtii MT* (De Hoff *et al.* 2013). We calculated frequencies of synonymous (d_S_) and nonsynonymous (d_N_) substitutions between gametologs for *G*. *pectorale MT* genes and found, with two exceptions, that they were even lower than in *C. reinhardtii* (Table S4 and Figure 3A) (Hiraide *et al.* 2013). These findings suggest either higher rates of gene conversion between *G. pectorale* gametologs than those in *C. reinhardtii*, and/or a younger age for its haplotype blocs that may have had less time to diverge. A potentially informative clue about haplotype divergence and the history of *G. pectorale MT* was revealed from one exceptional region at the distal end of *MT*– (bloc I in Figure 2A), which encompasses *TOC34* and part of neighboring gene *HSP70B* that resides in bloc II (Figure 2A). The genic regions of *TOC34* (d_N_ = 0.0623 and d_S_ = 0.5549; Figure 3B and Table S4), and part of *HSP70B* (d_N_ = 0.0006 and d_S_ = 0.0678; Figure 3C and Table S4) have up to 10-fold higher divergence values than all other gametolog pairs (Figure S4) (average d_N_ = 0.00333 ± 0.00288 and d_S_ = 0.0331 ± 0.0257), with a boundary in the middle of *HSP70B* where divergence between gametologs drops and resembles the remainder of bloc II (Figure 3C). High divergences are also evident in the intergenic regions around *TOC34* on both its proximal and distal sides relative to *HSP70B*, none of which can be aligned between the two haplotypes (Figure S2). By contrast, many of the intergenic sequences in blocs II and IV align well between haplotypes (Figure S2).

The discontinuity of divergences in the *TOC34-HSP70B* region has at least two possible explanations. One possibility is that the *TOC34-HSP70B* region is a “cold-spot” for recombination, and contains a sequence that blocks progression of gene conversion tracts near the middle of *HSP70B*. There is precedent for interhaplotype gene conversion in *C. reinhardtii* (De Hoff *et al.* 2013), though no isolated coldspots were found. A second possibility is that the highly similar regions of *G. pectorale MT* are relatively young, and that the more diverged region containing *TOC34* is a remnant from a previous version of *MT* with older and more diverged haplotypes. In this case, the low-divergence regions of *MT* could have arisen as a result of a replacement event, in which bloc II from one of the two *MT* haplotypes replaced the same region of the opposite mating haplotype (in inverted orientation) with a breakpoint between *TOC34* and *HSP70B* on one end, and between *WDR57* and *PTC1* on the other end (Figure S5). This resetting event might also have allowed renewed and continuing gene-conversion between gametologs in bloc II, but would leave *TOC34* gametologs in bloc I to continue diverging while also restricting the extent of gene conversion for *HSP70B* that abuts bloc I. The two explanations we propose for the discontinuous divergence patterns of bloc I *vs.* blocs II–IV of *G. pectorale MT* are not mutually exclusive, and in both cases they underscore the dynamic, and likely punctuated, nature of volvocine algal *MT* evolution.

### Characterization of GpFUS1

**Figure 4:**
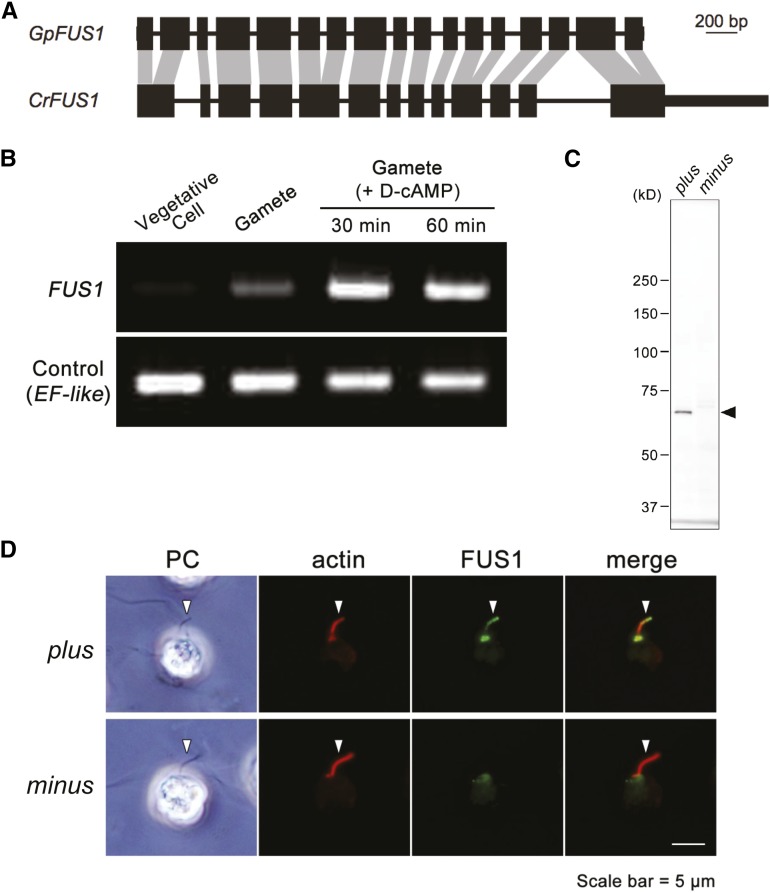
Identification and characterization of *GpFUS1*. (A) Comparison of *FUS1* intron–exon structures between *Chlamydomonas reinhardtii* and *Gonium pectorale*. Homologous CDS (black boxes) sequences are connected by gray shading. Introns are represented by thin lines. UTRs are represented by thick lines. (B) Semiquantitative RT-PCR of *GpFUS1* from *MT*+ *G*. *pectorale* strain. mRNAs were isolated from vegetative cells, gametes, and sexually activated gametes with db-cAMP (D-cAMP) treatment. *EF-like* gene was used as an internal control for RT-PCR. (C) Immunoblot analysis of GpFUS1 expression of activated plus and minus gametes using anti-GpFUS1 antibody. (D) Double immunofluorescence staining of db-cAMP activated mating type *plus* and *minus* gametes using anti-GpFUS1 and anti-actin antibody. Arrows indicate fertilization tubule, a mating apparatus that contains actin filament bundles.

The *G. pectorale FUS1* gene (*GpFUS1*) was identified based on a BLASTX search with the *G. pectorale MT* region queried against the *C. reinhardtii* proteome. Overall, *GpFUS1* has a similar intron–exon structure as *CrFUS1* (Figure 4A), with the addition of four additional introns not present in *CrFUS1*. The protein sequences of GpFUS1 and CrFUS1 have 31% amino acid identity, but retain the invasion/intimin repeats that may be involved in mediating mate recognition or membrane fusion (Figure S6 and Figure S7) (Misamore *et al.* 2003), and a signal peptide specifying membrane localization (Figure S8 and Figure S9).

*GpFUS1* mRNA expression was detected in gametes, but not in vegetative cells, and was upregulated in gametes by treatment with the mating-related signaling agonist db-cAMP (Figure 4B) (Mogi *et al.* 2012), similar to its ortholog *CrFUS1* (Pasquale and Goodenough 1987; Goodenough *et al.* 2007). Polyclonal antibodies against recombinant GpFUS1 recognized a protein on Western blots from *plus* but not *minus* gametes (Figure 4C). The anti-GpFUS1 signal (70 kDa) is smaller than the predicted molecular weight of GpFUS1 (∼90 kDa) in Western blotting analysis, suggesting that GpFUS1 might undergo post-translational processing. Immunofluorescence using the same antibodies localized GpFUS1 to the *plus* fertilization tubule in *G. pectorale* (Figure 4D) indicating a probable function in fertilization that is likely to be similar to that for CrFUS1; no signal was observed in the *minus* fertilization tubule.

Together, our results indicate that the *FUS1* gene has been conserved as a mating type *plus* gene in the volvocine lineage, with an ancestral function in fertilization that has persisted at least since the *G. pectorale–C. reinhardtii* split, and has been subsequently lost from the lineage leading to *V*. *carteri*. Currently, the *minus* gamete interacting partner for FUS1 is unknown, but our results predict that it will also be conserved between *C. reinhardtii* and *G. pectorale*.

### A dynamic history of MT locus evolution in the volvocine lineage

Previous comparative studies of volvocine algal *MT* loci have raised many questions about their evolutionary histories that this current study begins to address (Charlesworth and Charlesworth 2010; Umen 2011). The *C. reinhardtii* and *V. carteri MT* occupy a homologous chromosomal location, and share a common ancestral origin, but differ in many respects including gene content, gametolog divergence, locus size, syntenic bloc structure, and repeat content (Ferris *et al.* 2010). Although the *C. reinhardtii MT* locus is presumed to more closely reflect the ancestral state of volvocine algal *MT* loci than the *V. carteri MT* locus, without additional information about *MT* loci from other volvocine species, the evolutionary trajectory of the mating type region cannot be unambiguously inferred. The structure and gene content of *G. pectorale MT* described here begins to clarify the evolutionary history of volvocine algal *MT* loci. In overall size, structure, and gametolog divergence, *G. pectorale MT* resembles *C. reinhardtii MT*: it has just a few syntenic blocs containing multiple genes (Figure 2 and Figure S2), low divergence between most gametolog pairs (Figure 3A), and alignable intergenic regions interrupted in just a few positions with repeats (Figure S2). It also resembles *C. reinhardtii* in its complement of key mating regulators: *MID* and *MTD1* in *MT*–, and *FUS1* in *MT+*. However, *G. pectorale MT* differs from *C. reinhardtii MT* in its apparent lack of autosomal insertions into the *MT* region, and in the composition of its gametologs, which have more in common with those in *V. carteri MT* (Figure 1 and Table S3). Intriguingly, a small region of *G*. *pectorale MT* in the *TOC34-HSP70B* region shows elevated divergence between gametologs, which approaches divergence levels found for *V. carteri* gametologs, and may represent an early stage of *MT* differentiation in this lineage (Figure 3 and Figure S5).

**Figure 5:**
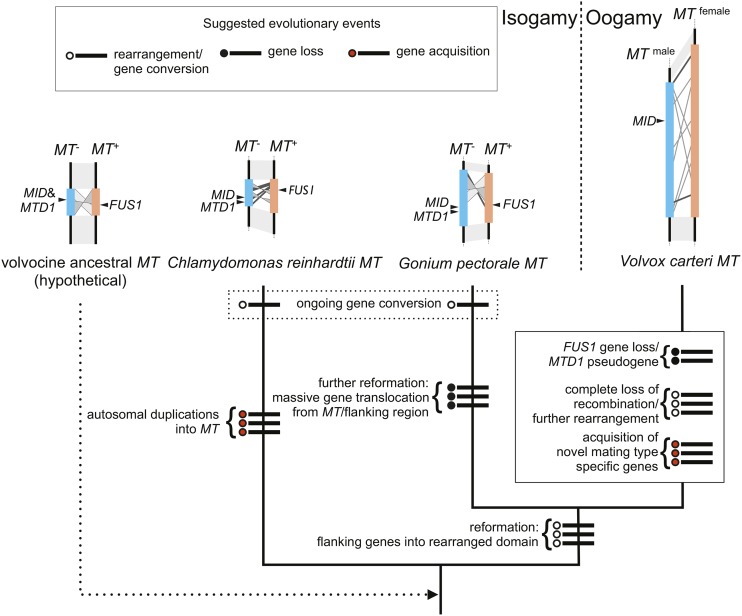
Possible evolutionary history for volvocine *MT* loci based on minimal changes necessary to explain observed results in this study and in previous studies (Ferris *et al.* 2002, 2010; De Hoff *et al.* 2013). Each proposed event is indicated by a thick line crossing the node accompanying a circle: open, rearrangement or gene conversion event; filled, gene loss; red, gene acquisition.

Based on these similarities and differences, we can infer a minimal set of changes that led to the generation of three distinct mating locus regions of *C. reinhardtii*, *G. pectorale*, and *V. carteri* (Figure 5). While the actual history of this region is likely to be more complicated than in our depiction, and awaits validation, our model creates a framework for classifying the types of changes that occurred in volvocine *MT* loci, and possible ancestral states. The common ancestor of all volvocine algae most likely had a mating locus near its telomere on a chromosome that is syntenic with Chromosome 6 in *C. reinhardtii*. The ancestral *MT* locus would have contained *MID* and *MTD1* homologs in its *MT*– haplotype, and a *FUS1* homolog in its *MT+* haplotype. Its gametologs were genes found on the current *C. reinhardtii* Chromosome 6, or *V. carteri* Linkage Group I. After the *G. pectorale* and *C. reinhardtii* lineages split, one or both of their *MT* loci underwent a major reconfiguration, in which a different group of gametologs became fixed around the rearranged region of *MT*. During this time, the process of gametolog gene conversion was ongoing, and maintained relative homogeneity between gametolog pairs between *plus* and *minus* haplotypes in each lineage. The autosomal insertions in *C. reinhardtii MT+* (De Hoff *et al.* 2013) were probably not lost in the *G. pectorale* lineage, but gained in the *C. reinhardtii* lineage, although the latter possibility cannot be excluded (Figure 5). In either case, the appearance of *G. pectorale MT* is “younger” and less complex than that of *C. reinhardtii*, which suggests a recent reconfiguration. Supporting the idea of reconfiguration is our finding of a small, and possibly more ancient, region containing *TOC34* and part of *HSP70B* that underwent substantial differentiation (Figure 3 and Figure S2). By the time of the split between the *G. pectorale* and *V. carteri* lineages, their common ancestor had acquired many of the gametologs that are still common between these two species. We cannot determine when the *FUS1* gene was lost from the *V*. *carteri* lineage, but the *MTD1* gene may have been lost relatively recently, as a possible *MTD1* pseudogene was identified in the male *V. carteri MT* haplotype (Ferris *et al.* 2010). In addition, *V. carteri MT* gained over a dozen male-limited or female-limited *MT* genes that lack detectable homologs in *G. pectorale* or elsewhere, suggesting that these additional genes arose after the two lineages split. At some point after the *G. pectorale-V. carteri* split, gametolog divergence in the *V. carteri* accelerated, and the R domain of its *MT* locus underwent a large expansion. Although we do not know when accelerated evolution and expansion began in the *V*. *carteri* lineage, there is additional information on gametolog divergence for the *MT* gene *MAT3*, whose sequences have been identified in *P*. *starrii*, an oogamous colonial species that is more closely related to *V. carteri* than to *G. pectorale* (Figure 1). While the male and female *MAT3* gametologs of *V. carteri* are highly diverged (Ferris *et al.* 2010), those in *P. starrii* have a low divergence that is comparable to that found in *G. pectorale* (Hiraide *et al.* 2013). If the *P. starrii MAT3* data are representative of its *MT* locus as a whole, then the massive divergence observed for *V. carteri MT* gametologs occurred relatively recently in the lineage.

### Conclusion

While many questions remain about the history, timing, and mechanism of volvocine algal *MT* evolution, our data begin to elucidate the highly dynamic nature of their diversification, and to establish the trajectory of this process. Overall, there appears to be relative stasis in terms of *MT* chromosomal location, and in the presence of the sex-determining gene *MID*. *FUS1* and *MTD1* appear to have persisted in a large part of the volvocine lineage, but became dispensable in *V. carteri*, and perhaps its close relatives. While *MTD1* is not totally essential for mating-type differentiation in *C. reinhardtii*, *FUS1* is essential for fertilization (Goodenough *et al.* 2007; Lin and Goodenough 2007). How and why the *FUS1* function was dispensed with or replaced in *V. carteri* (and perhaps other volvocine species), and how this change relates to its sexual cycle are interesting areas for future investigation.

It is unclear why *MT* remains associated with the same chromosome in the three widely separated volvocine species where it has been characterized, but is suggestive that interchromosomal sequence exchanges are much less frequent than intrachromosomal exchanges and rearrangements. Large changes in *MT* haplotype structure, gametolog content, and gametolog divergence have all occurred in volvocine algae. In all these areas, there may be episodic upheavals where the *MT* locus structure is reconfigured, and where the competing effects of autosomal insertions, gametolog gene conversion, and recombination suppression shape haplotype differentiation. Data on haploid mating-type, or sex chromosome evolution, from other lineages suggests that processes similar to those we have documented here for volvocine algae are likely to take place elsewhere. For example, gene conversion between gametologs has been documented in fungal mating loci, as have rearrangements and haplotype bloc formation associated with recombination suppression (Sun *et al.* 2012). Much less is known about haploid SDR regions outside of fungi, although some data are now available for haploid bryophyte sex chromosomes (Yamato *et al.* 2007; McDaniel *et al.* 2013), and for the SDR region of the brown alga *Ectocarpus* sp. (Ahmed *et al.* 2014). Future comparative studies in these other groups, and within the volvocine lineage, will help elucidate the mechanisms that contribute to mating locus and sex chromosome evolution, and how these mechanisms impact the evolution of sex and sexual cycles.

## Supplementary Material

Supplemental Material
