# Supplementary material for: Sequence of the Gonium pectorale Mating Locus Reveals a Complex and Dynamic History of Changes in Volvocine Algal Mating Haplotypes
Source: G3 (Bethesda). 2016 Feb 22;6(5):1179–89. doi: 10.1534/g3.115.026229 (PMC4856071; doi:10.1534/g3.115.026229)
Supplement: Supplemental Material [file supp_6_5_1179__index.html]

Sequence of the Gonium pectorale Mating Locus Reveals a Complex and Dynamic History of Changes in Volvocine Algal Mating Haplotypes — Supplemental Material 

# Sequence of the *Gonium pectorale* Mating Locus Reveals a Complex and Dynamic History of Changes in Volvocine Algal Mating Haplotypes

## Supplemental Material for Hamaji *et al.*, 2016

**Files in this Data Supplement:**

- Figure S1 - BAC assembly overview of *Gonium pectorale MT* locus. (.pdf, 227 KB)
- Figure S2 - Dot plot comparison of *Gonium pectorale MT*- (horizontal) and *MT*+ (vertical): sequences with blue, aligned in forward orientation; red, reverse. (.pdf, 220 KB)
- Figure S3 - Schematic diagram of syntenic relationships between the *MT* scaffold and the nearby related scaffold00010 of *Gonium pectorale* and chromosome 6 of *Chlamydomonas reinhardtii*. (.pdf, 160 KB)
- Figure S4 - Sliding window analyses of *Gonium pectorale* gametologs. (.pdf, 542 KB)
- Figure S5 - A model for the observed discontinuity in sequence divergence and structure of *G. pectorale MT*. (.pdf, 70 KB)
- Figure S6 - Alignment of the amino acid sequences of the GpFUS1 and CrFUS1 proteins. (.pdf, 697 KB)
- Figure S7 - Invasin/intimin immunoglobulin (Ig)-like repeats found in both *Chlamydomonas reinhardtii* and *Gonium pectorale* FUS1 based on Misamore et al. (2003). (.pdf, 144 KB)
- Figure S8 - Comparison of FUS1 hydrophobicity. (.pdf, 169 KB)
- Figure S9 - Schematic comparison of structure predictions for FUS1 proteins. (.pdf, 175 KB)
- Table S1 - Primers used in this study. (.pdf, 52 KB)
- Table S2 - Presence or absence of mating type/sex specifically coded genes in volvocine *MT* loci. (.pdf, 64 KB)
- Table S3 - Locations of *Volvox carteri* and *Chlamydomonas reinhardtii MT* R domain genes in *Gonium pectorale.* (.pdf, 107 KB)
- Table S4 - Non-synonymous (dN) and synonymous (dS) substitutions of gametologs in *Gonium pectorale*. (.pdf, 56 KB)
