# Supplementary material for: Sequence of the Gonium pectorale Mating Locus Reveals a Complex and Dynamic History of Changes in Volvocine Algal Mating Haplotypes
Source: G3 (Bethesda). 2016 Feb 22;6(5):1179–89. doi: 10.1534/g3.115.026229 (PMC4856071; doi:10.1534/g3.115.026229)
Supplement: Supplemental Material [file supp_g3.115.026229_FigureS1.pdf]

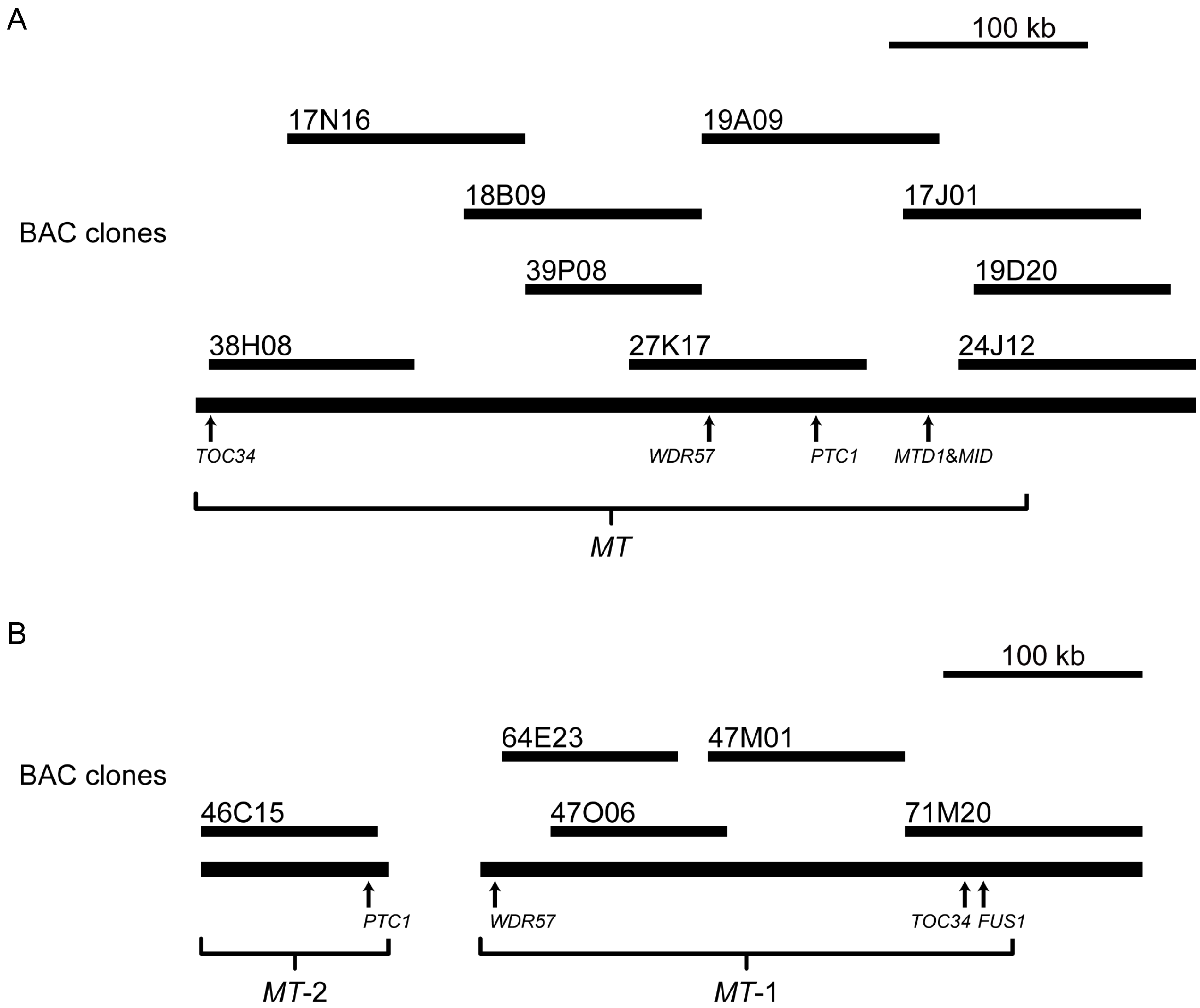

Figure S1. BAC assembly overview of *Gonium pectorale MT* locus. BAC clones identified and sequenced for tiling are shown above (A: *minus*; B: *plus*). The putative “rearranged” *MT* regions are marked with *MT* (*minus* R domain), *MT*-1 and *MT*-2 (plus R domain). Further scaffolding based on Illumina HiSeq/MiSeq paired-end and PacBio sequencing connected *MT*-1 and -2 as in Figure 1 (i.e. *PTC1* as distal from *WDR57*).
