# Supplementary material for: Sequence of the Gonium pectorale Mating Locus Reveals a Complex and Dynamic History of Changes in Volvocine Algal Mating Haplotypes
Source: G3 (Bethesda). 2016 Feb 22;6(5):1179–89. doi: 10.1534/g3.115.026229 (PMC4856071; doi:10.1534/g3.115.026229)
Supplement: Supplemental Material [file supp_g3.115.026229_FigureS2.pdf]

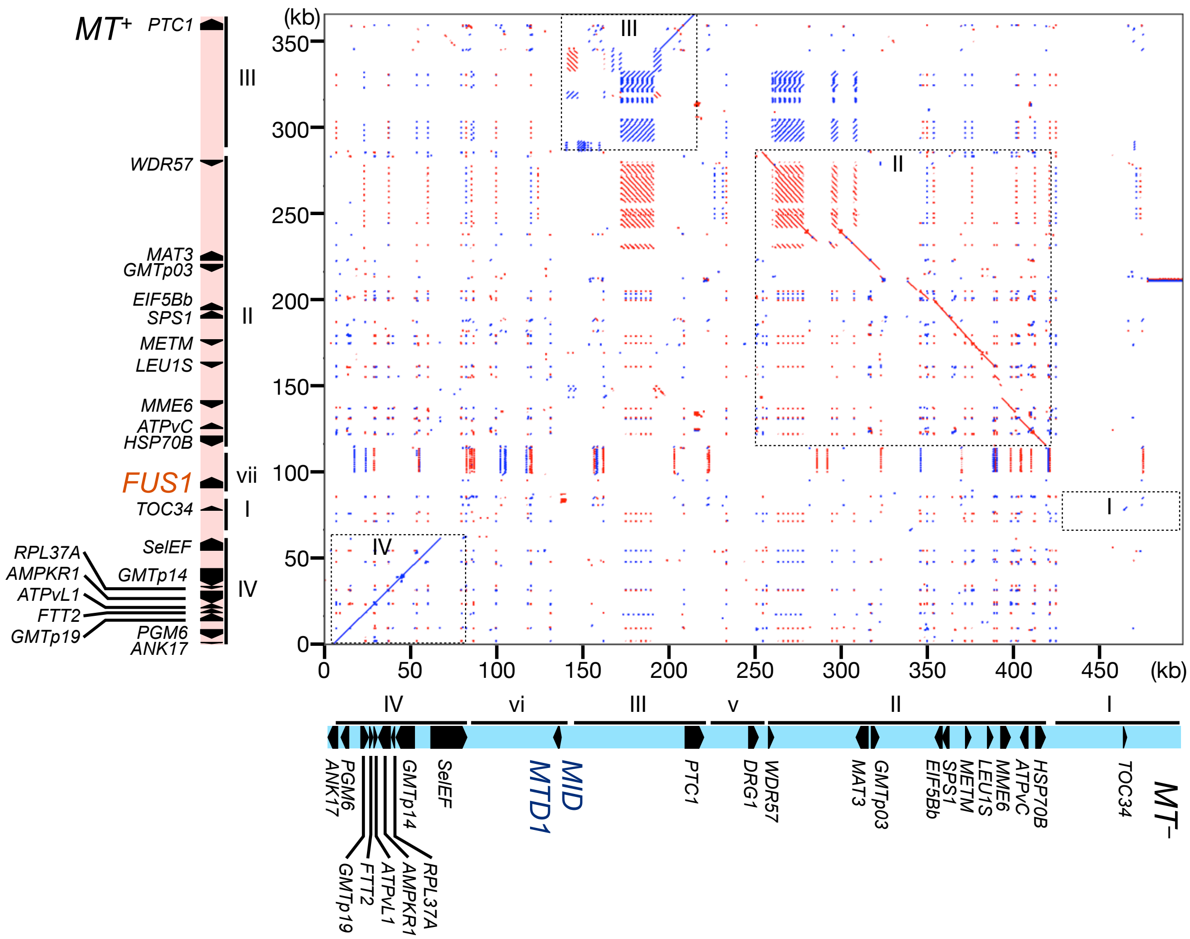

Figure S2. Dot plot comparison of *Gonium pectorale MT*- (horizontal) and *MT*+ (vertical): sequences with blue, aligned in forward orientation; red, reverse. Bloc designations are corresponding to Figure 2. Syntenic blocs I-IV are bounded by dashed rectangles.
