# Supplementary material for: Sequence of the Gonium pectorale Mating Locus Reveals a Complex and Dynamic History of Changes in Volvocine Algal Mating Haplotypes
Source: G3 (Bethesda). 2016 Feb 22;6(5):1179–89. doi: 10.1534/g3.115.026229 (PMC4856071; doi:10.1534/g3.115.026229)
Supplement: Supplemental Material [file supp_g3.115.026229_FigureS3.pdf]

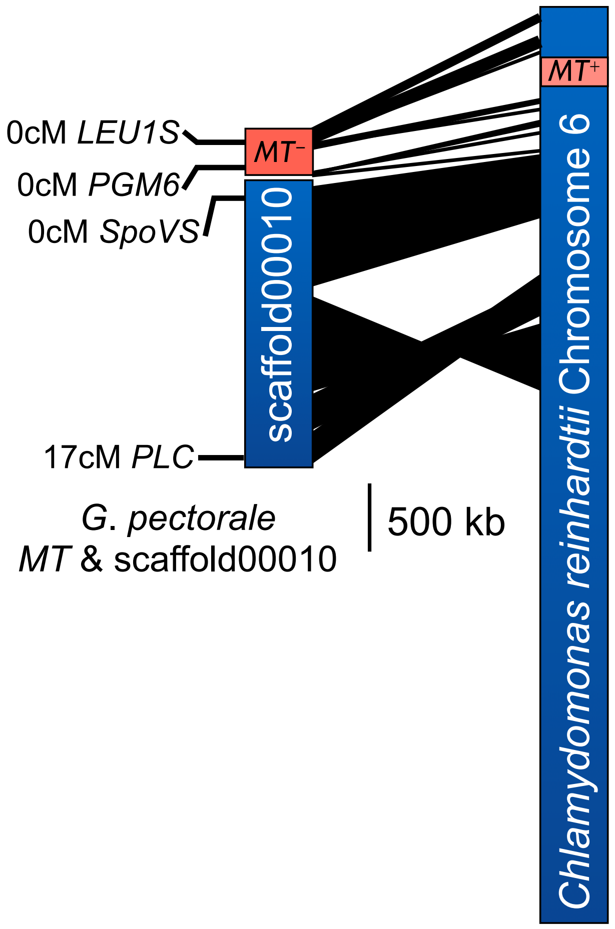

Figure S3. Schematic diagram of syntenic relationships between the *MT* scaffold and the nearby related scaffold00010 of *Gonium pectorale* and chromosome 6 of *Chlamydomonas reinhardtii*. The linkage of genetic markers to the mating phenotype on *G. pectorale* is indicated on the left of its scaffold.
