# Supplementary material for: Sequence of the Gonium pectorale Mating Locus Reveals a Complex and Dynamic History of Changes in Volvocine Algal Mating Haplotypes
Source: G3 (Bethesda). 2016 Feb 22;6(5):1179–89. doi: 10.1534/g3.115.026229 (PMC4856071; doi:10.1534/g3.115.026229)
Supplement: Supplemental Material [file supp_g3.115.026229_FigureS4.pdf]

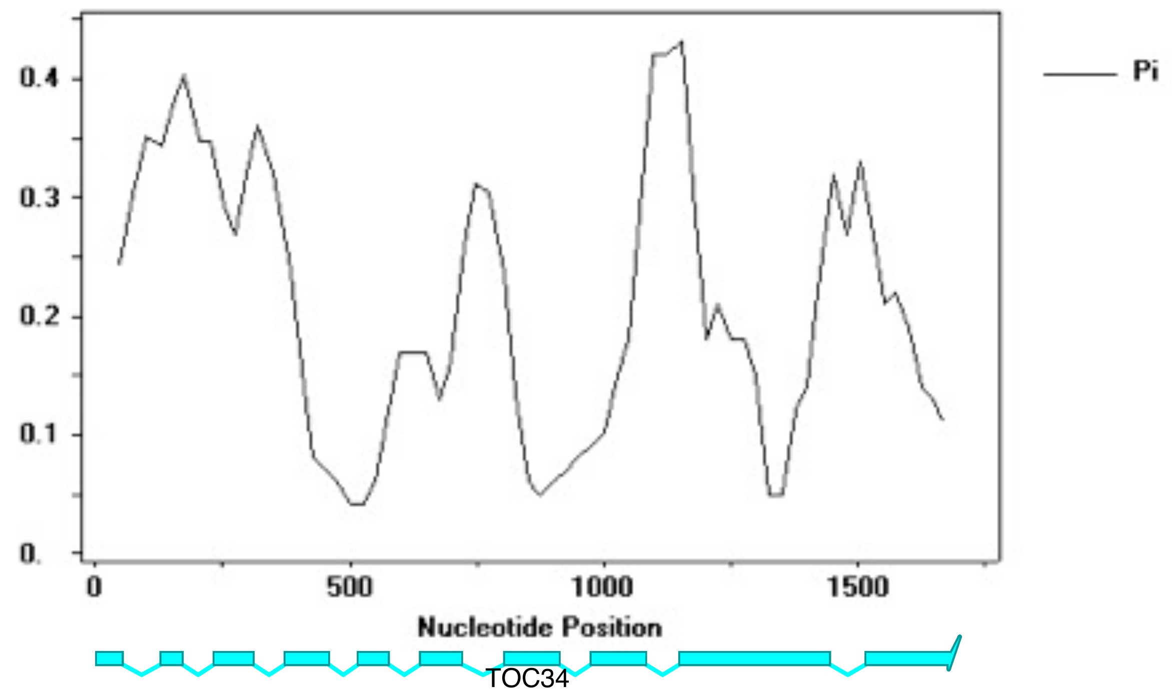

Pi
0.4
0.3
0.2
0.1
0.
0
500
1000
1500
Nucleotide Position
TOC34

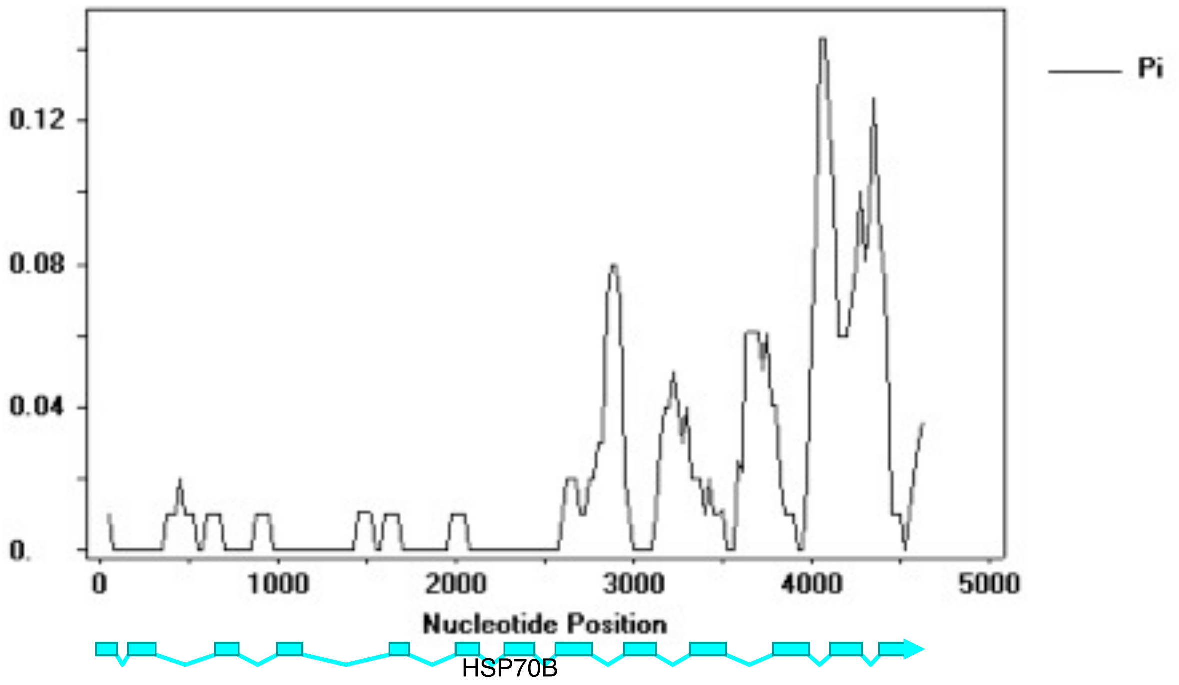

Pi
0.12
0.08
0.04
0.
0
1000
2000
3000
4000
5000
Nucleotide Position
HSP70B

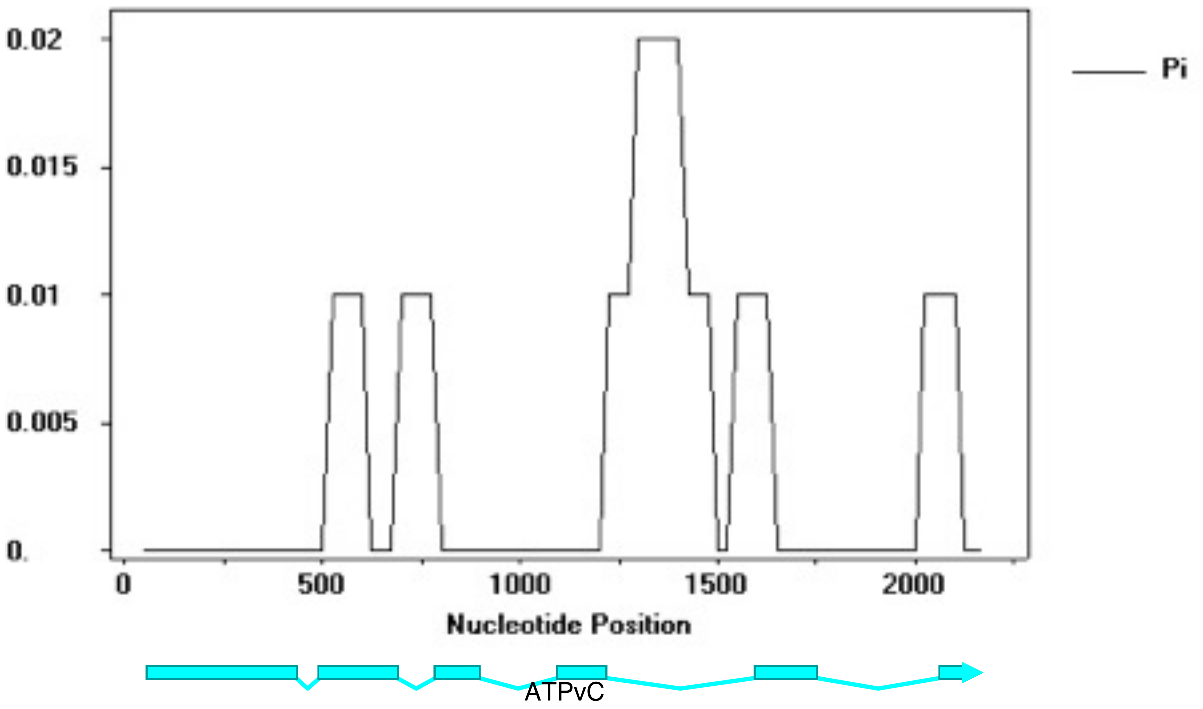
0.02
0.015
0.01
0.005
0.
0
500
1000
1500
2000
Nucleotide Position
Pi
ATPvC

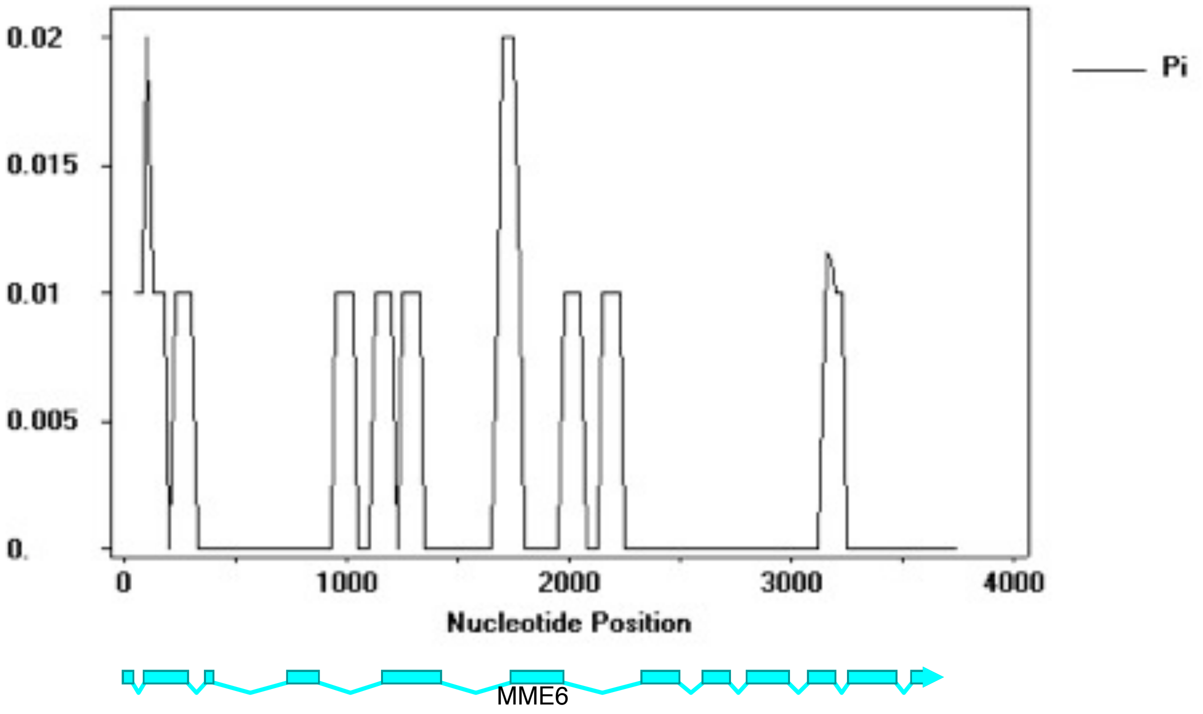
0.02
0.015
0.01
0.005
0.
0
1000
2000
3000
4000
Nucleotide Position
Pi
MME6

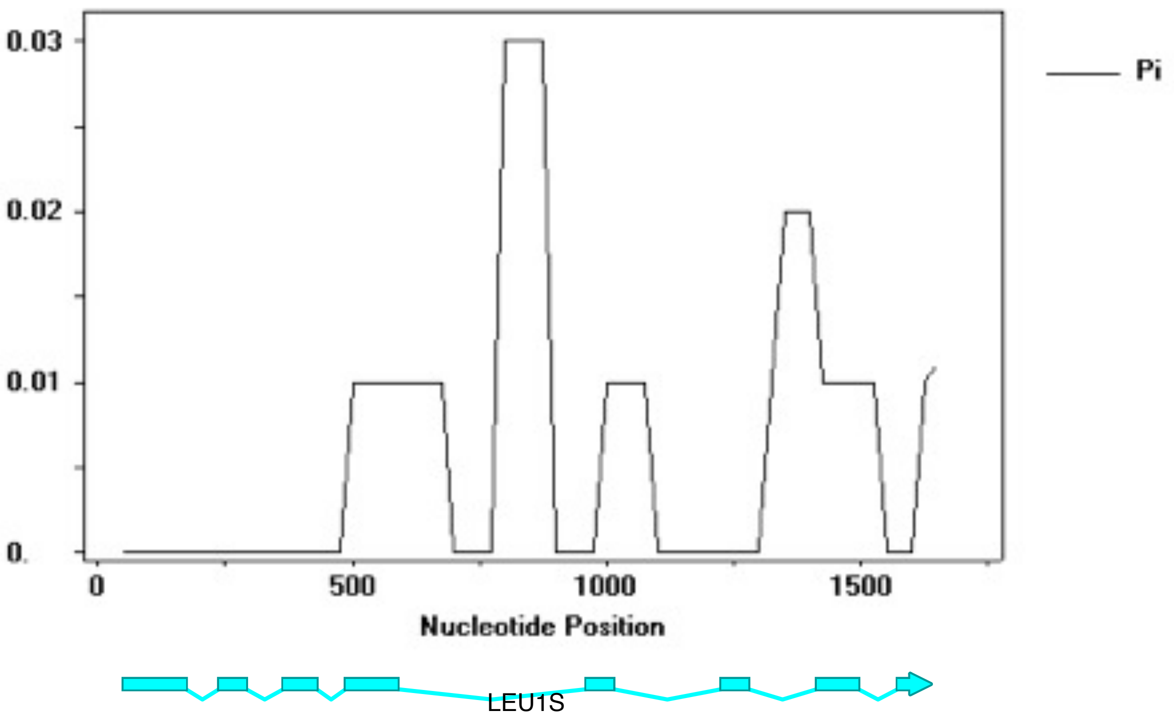
0.03
0.02
0.01
0.
0
500
1000
1500
Nucleotide Position
Pi
LEU1S

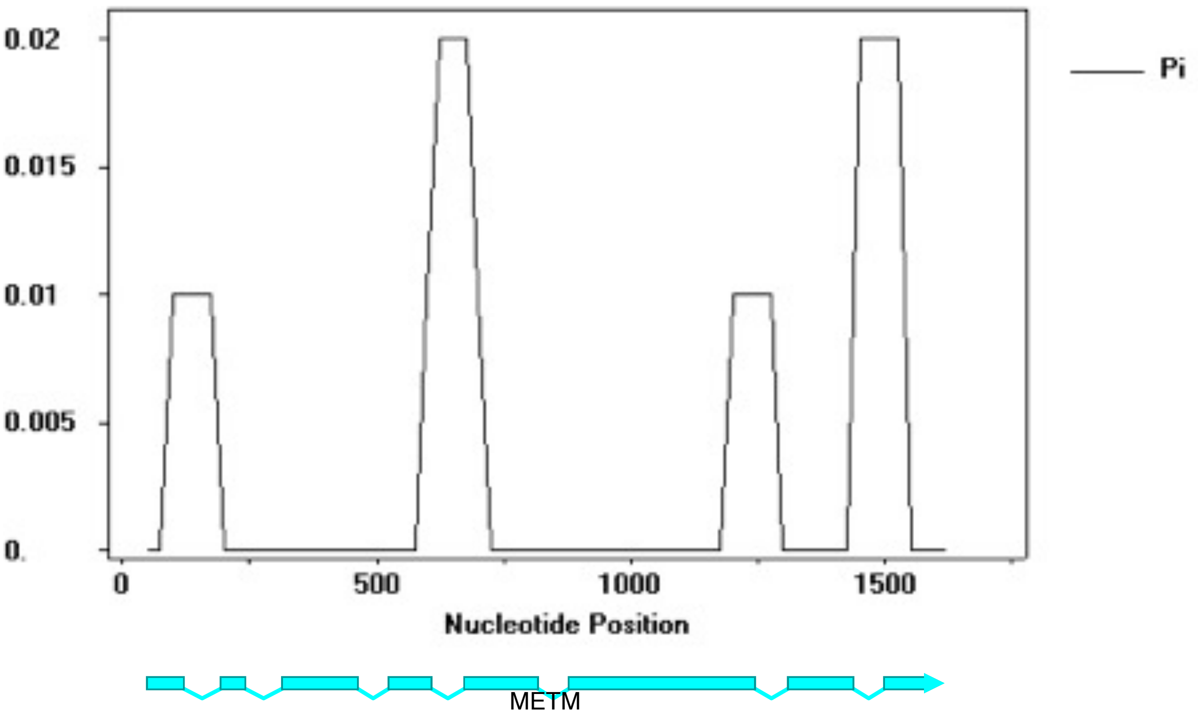
0.02
0.015
0.01
0.005
0.
0
500
1000
1500
Nucleotide Position
Pi
METM

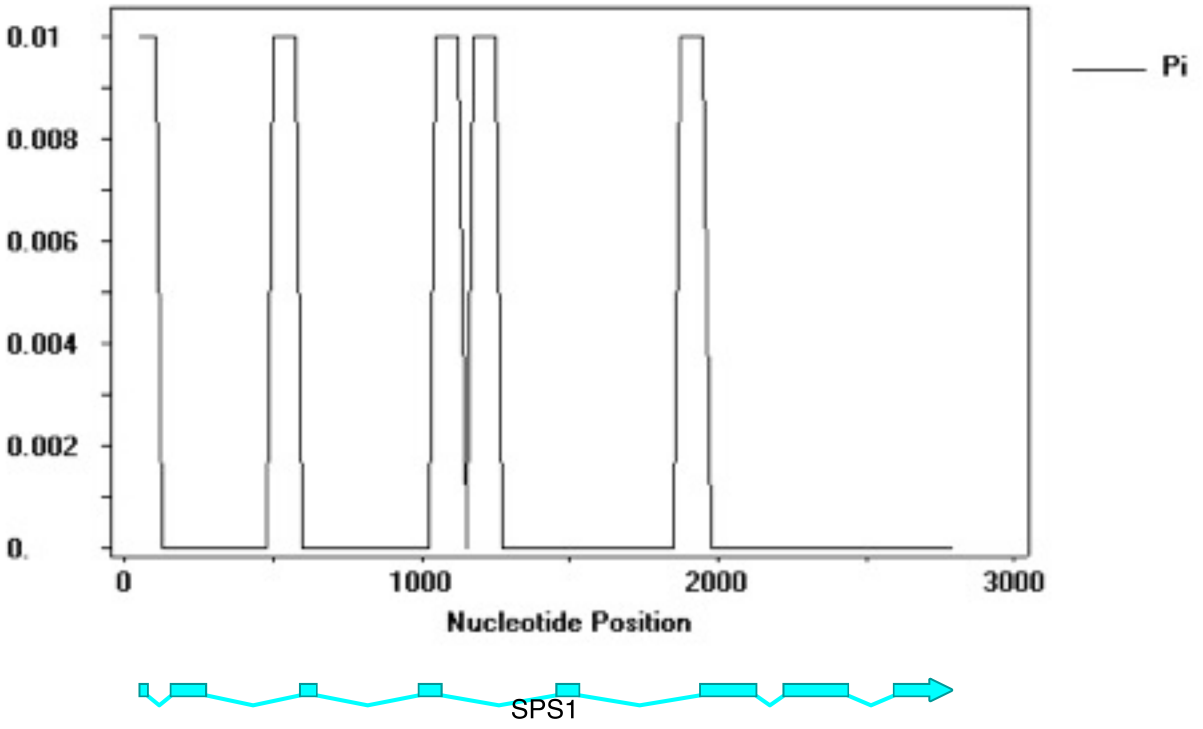
0.01
0.008
0.006
0.004
0.002
0.
0
1000
2000
3000
Nucleotide Position
Pi
SPS1

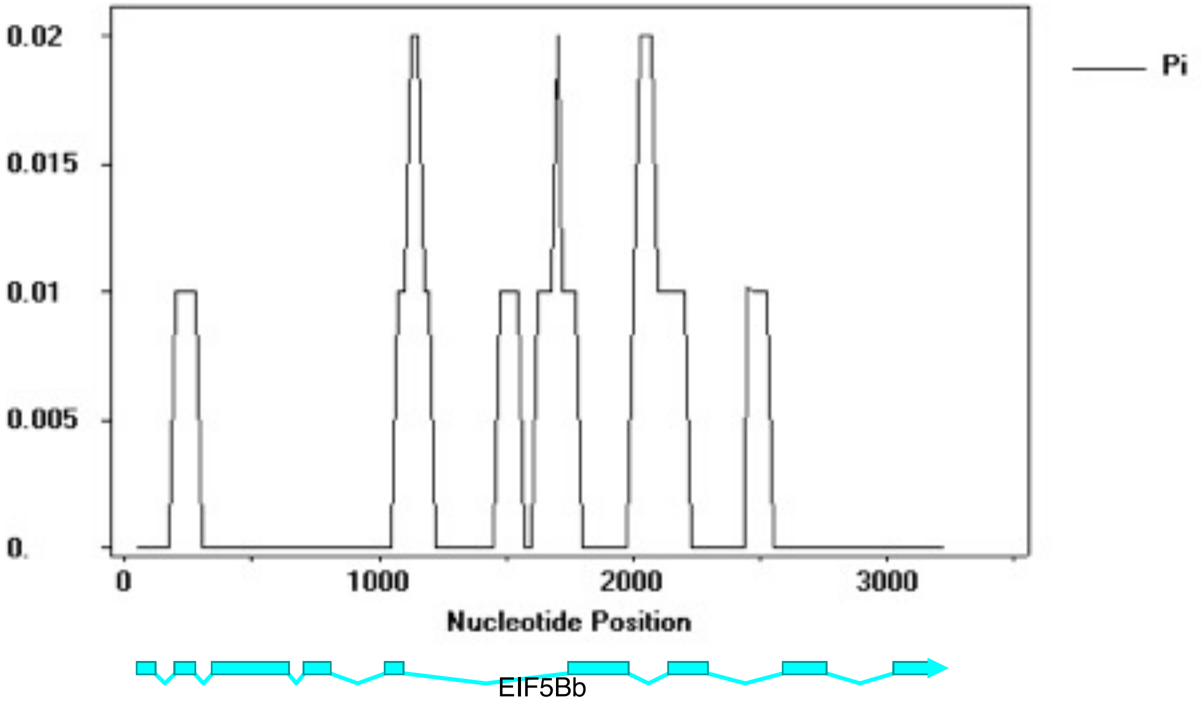
0.02
0.015
0.01
0.005
0.
0
1000
2000
3000
Nucleotide Position
Pi
EIF5Bb

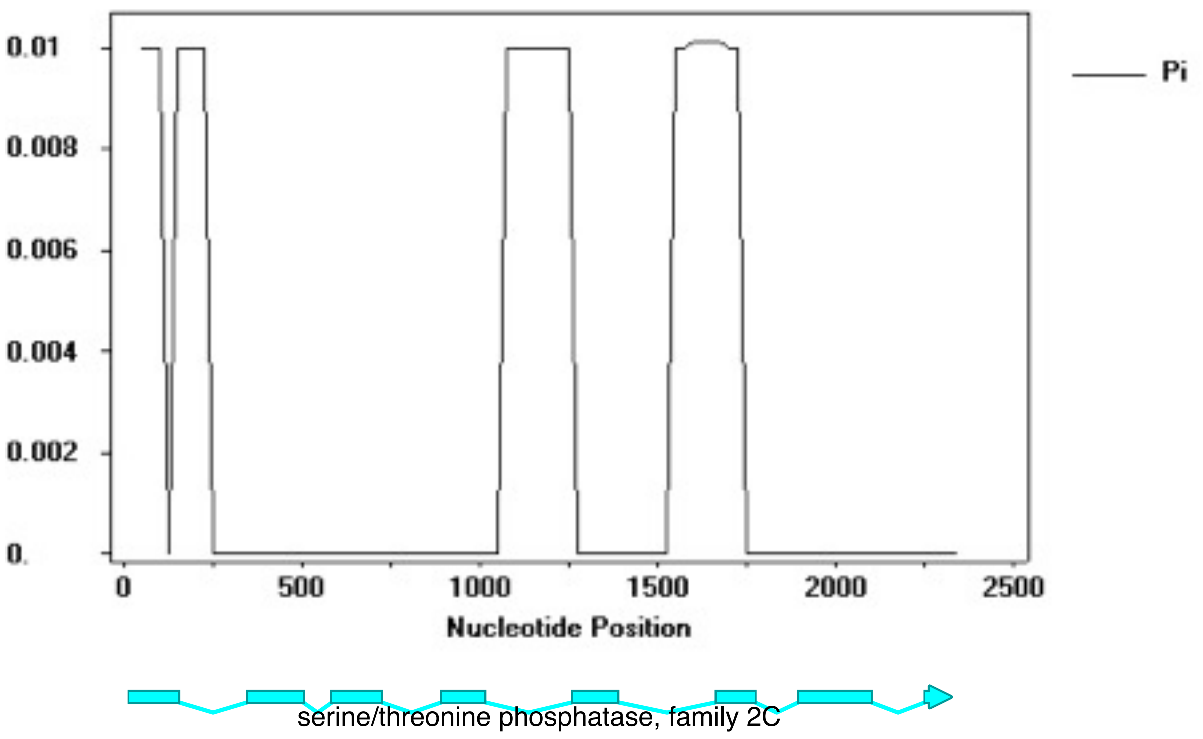
0.01
0.008
0.006
0.004
0.002
0.
0
500
1000
1500
2000
2500
Nucleotide Position
Pi
serine/threonine phosphatase, family 2C

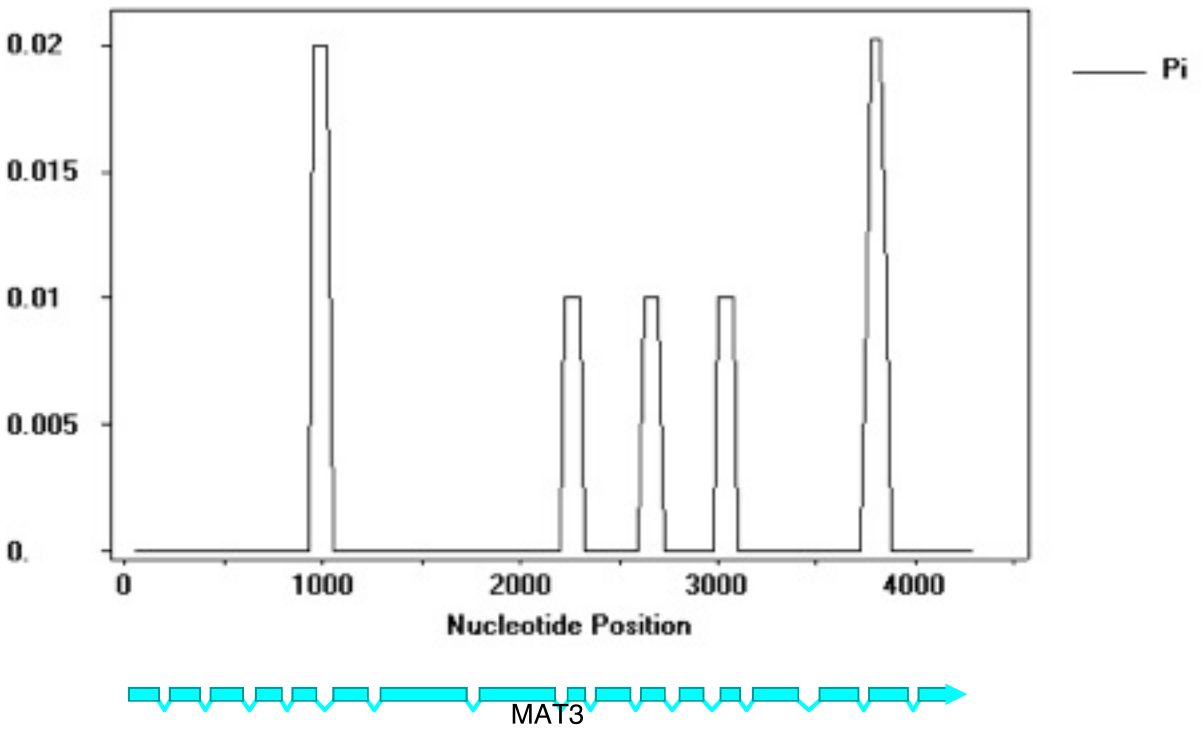
0.02
0.015
0.01
0.005
0.
0
1000
2000
3000
4000
Nucleotide Position
Pi
MAT3

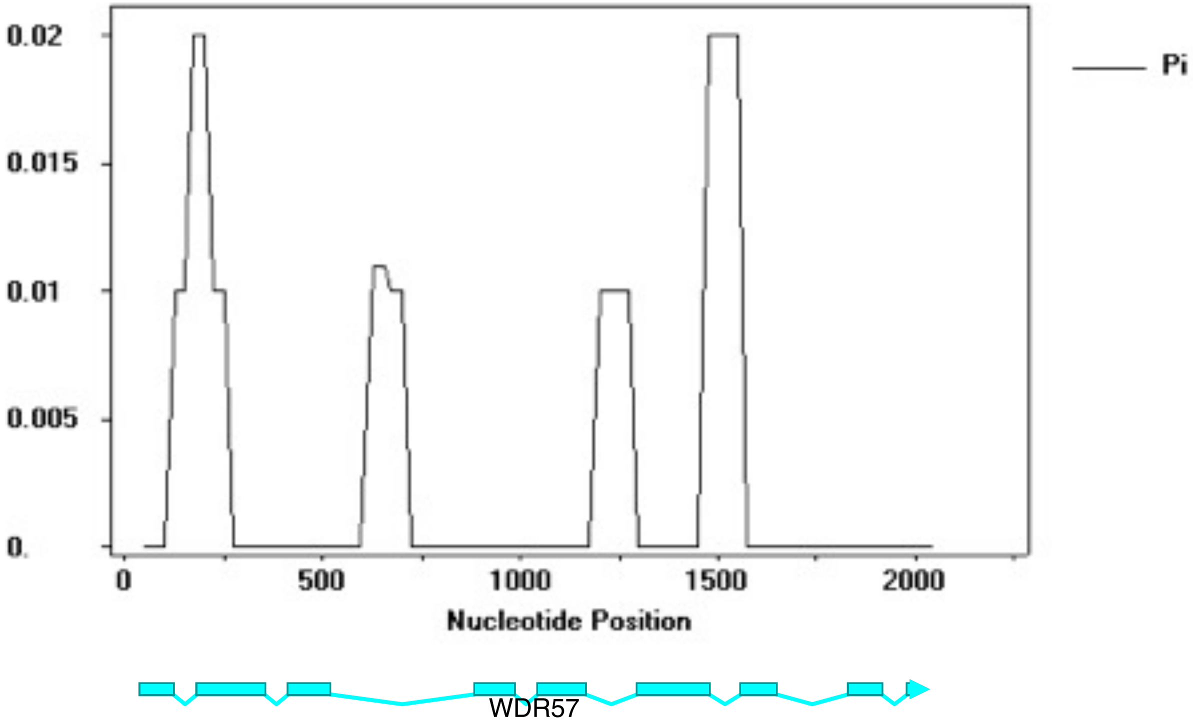
0.02
0.015
0.01
0.005
0.
0
500
1000
1500
2000
Nucleotide Position
Pi
WDR57

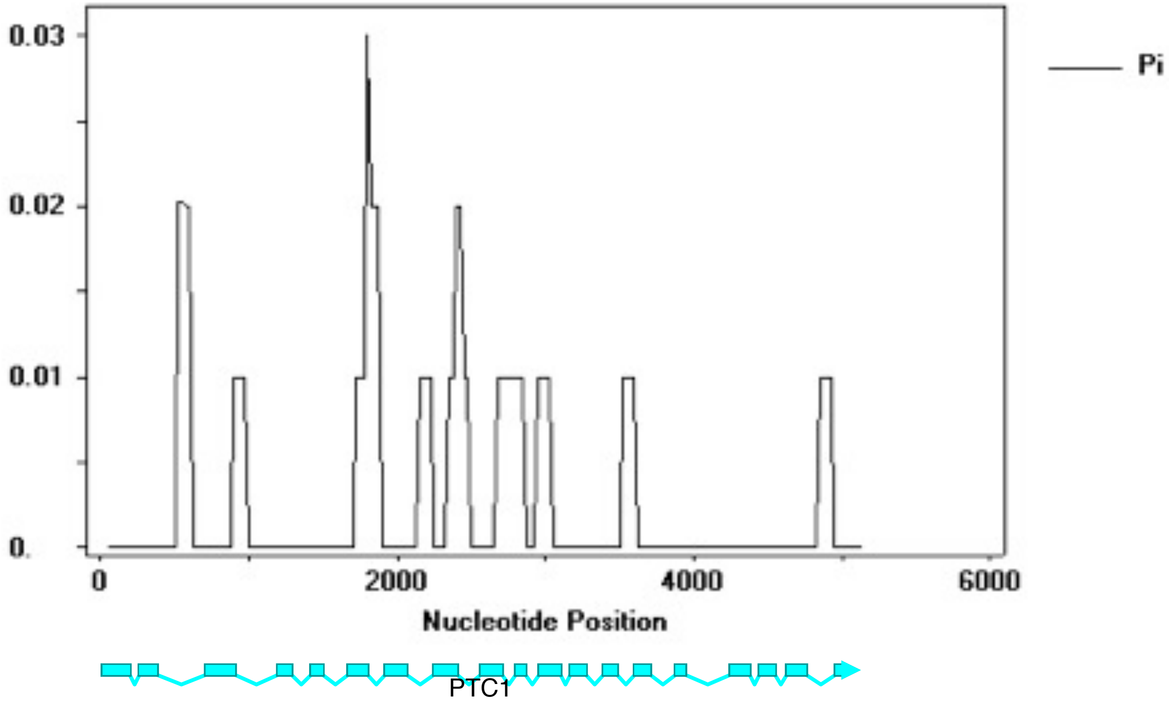
0.03
0.02
0.01
0.
0
2000
4000
6000
Nucleotide Position
Pi
PTC1

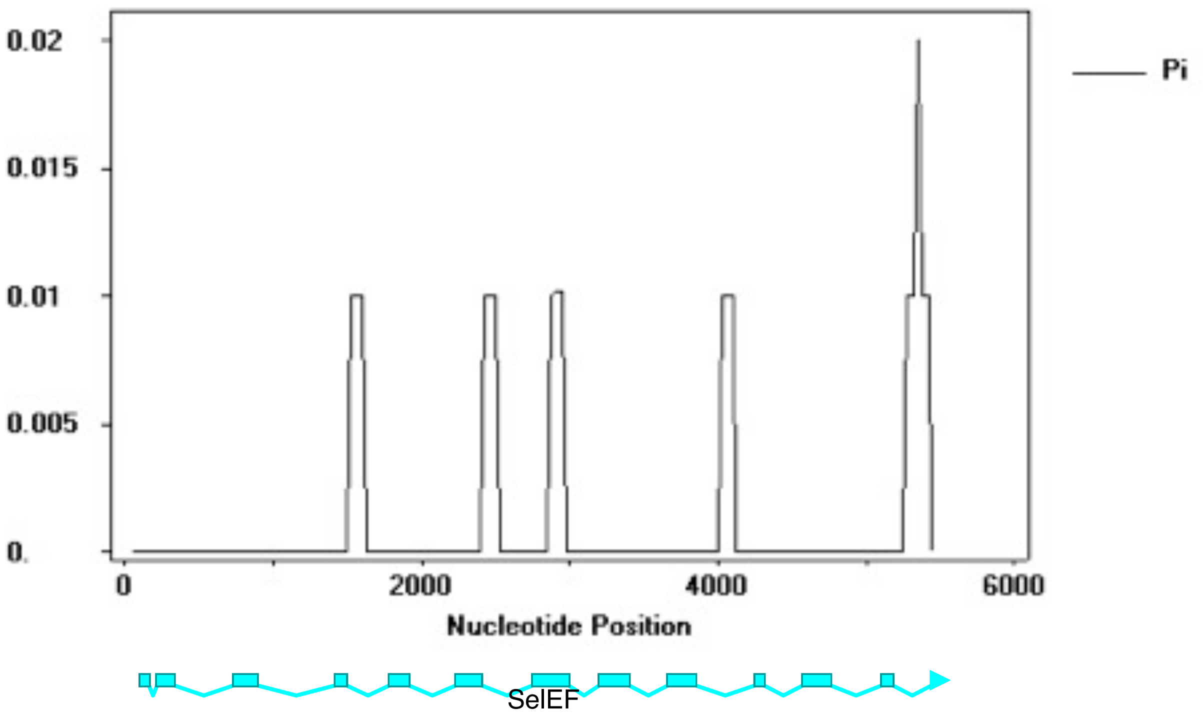
0.02
0.015
0.01
0.005
0.
0
2000
4000
6000
Nucleotide Position
Pi
SelEF

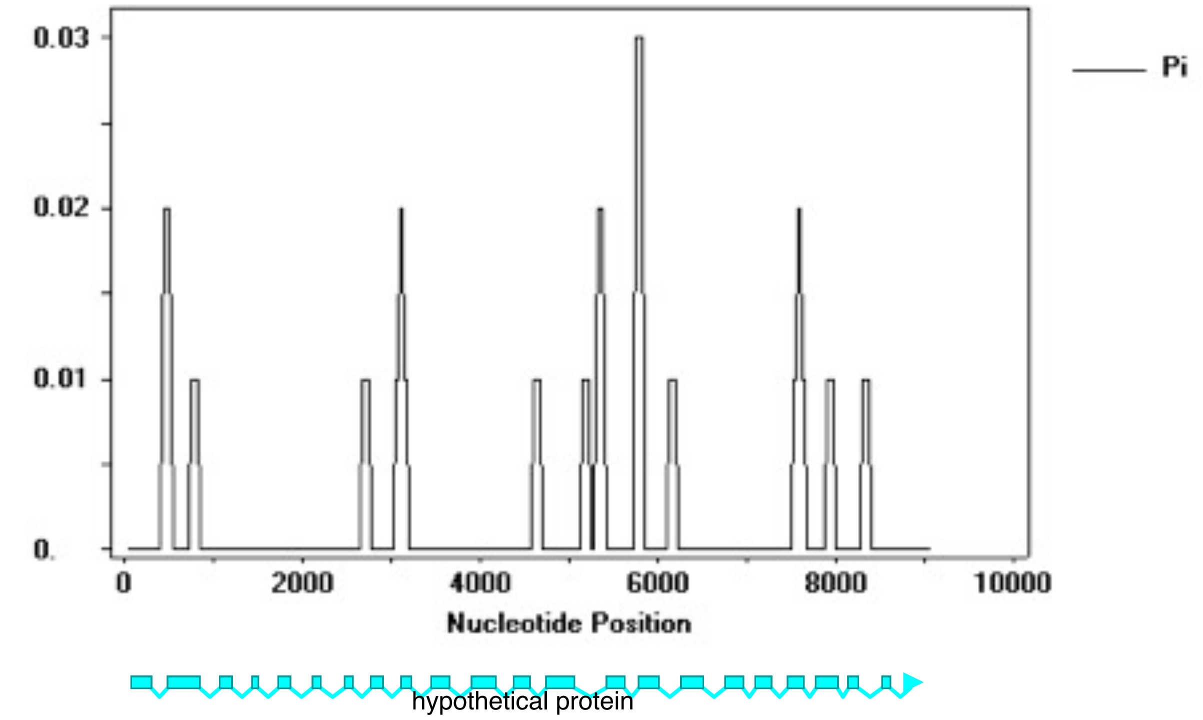
0.03
0.02
0.01
0.
0
2000
4000
6000
8000
10000
Nucleotide Position
Pi
hypothetical protein

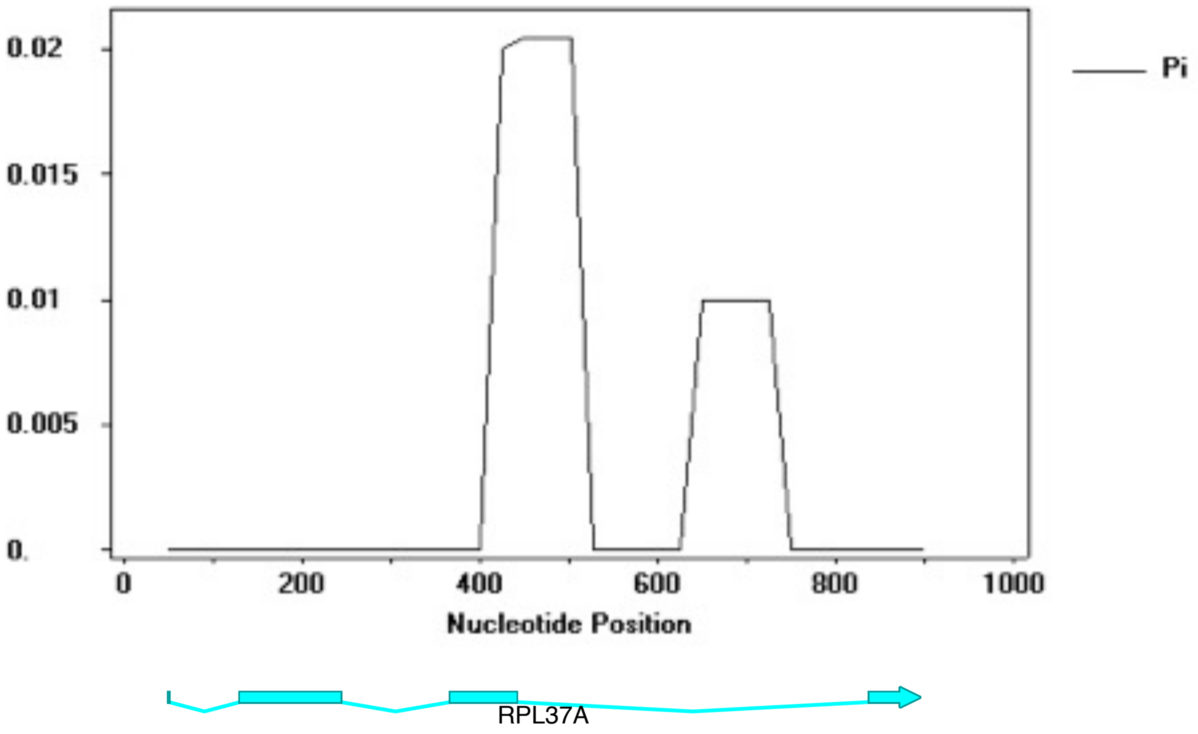
0.02
0.015
0.01
0.005
0.
0
200
400
600
800
1000
Nucleotide Position
Pi
RPL37A

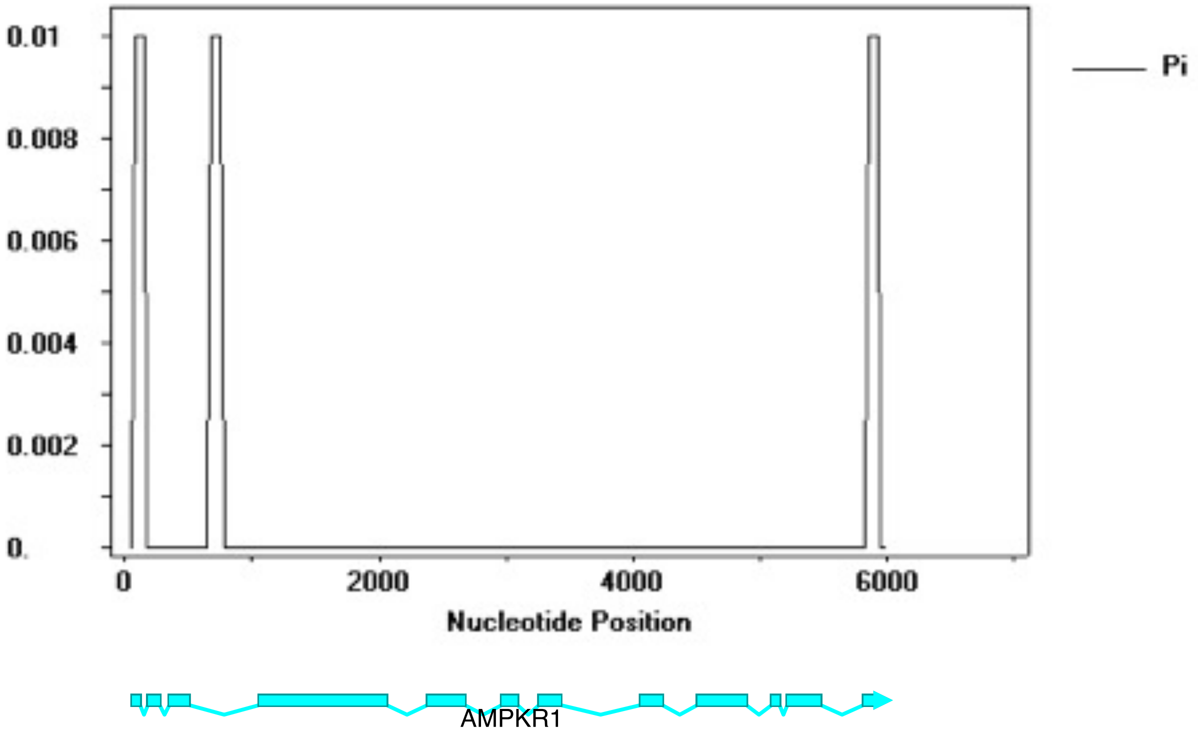
0.01
0.008
0.006
0.004
0.002
0.
0
2000
4000
6000
Nucleotide Position
Pi
AMPKR1

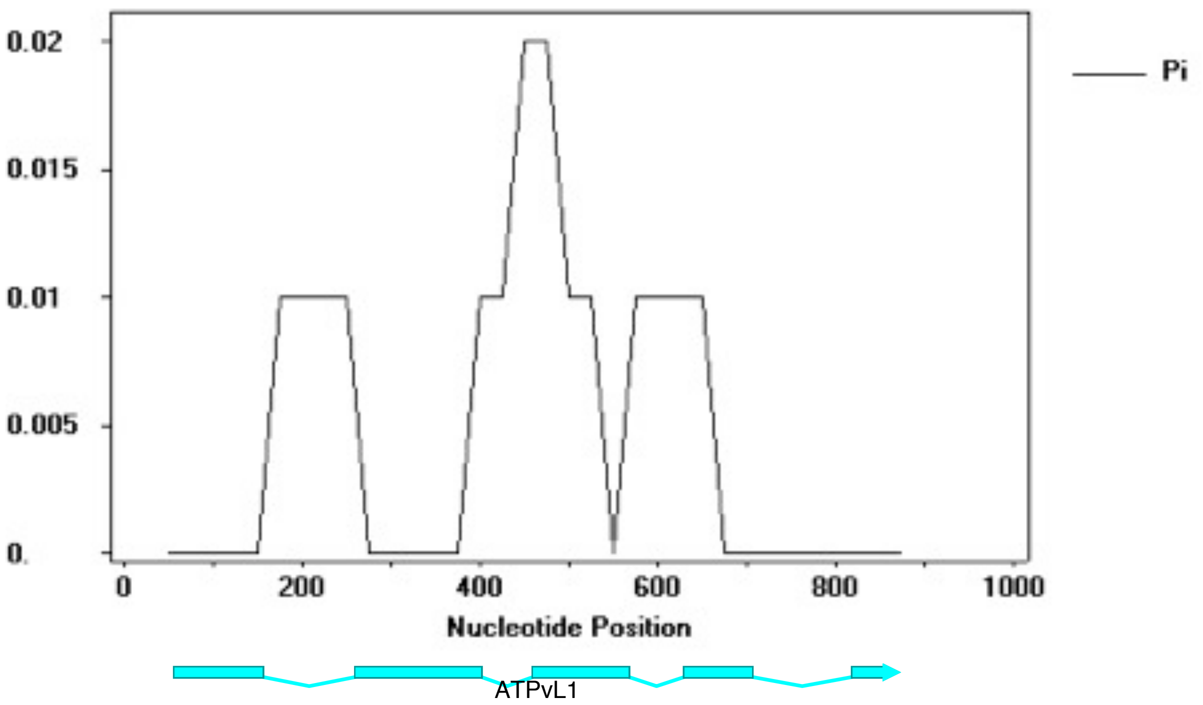

0.02
0.015
0.01
0.005
0.
0
200
400
600
800
1000
Nucleotide Position
Pi
ATPvL1

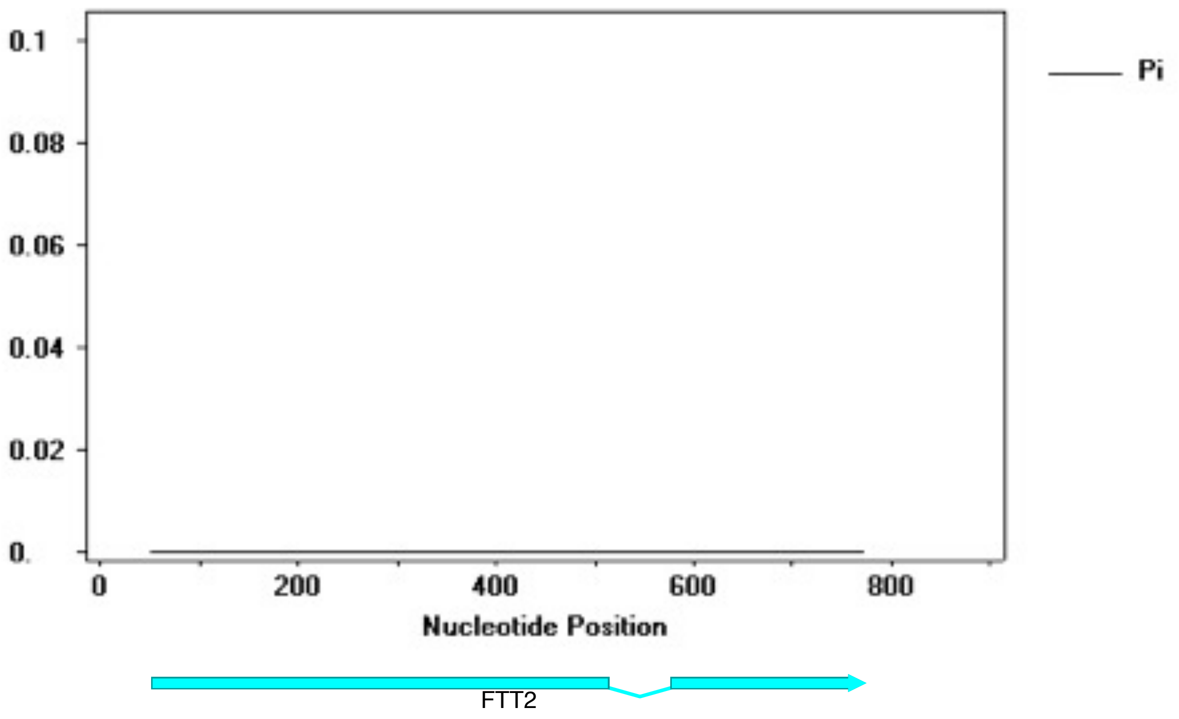

0.1
0.08
0.06
0.04
0.02
0.
0
200
400
600
800
Nucleotide Position
Pi
FTT2

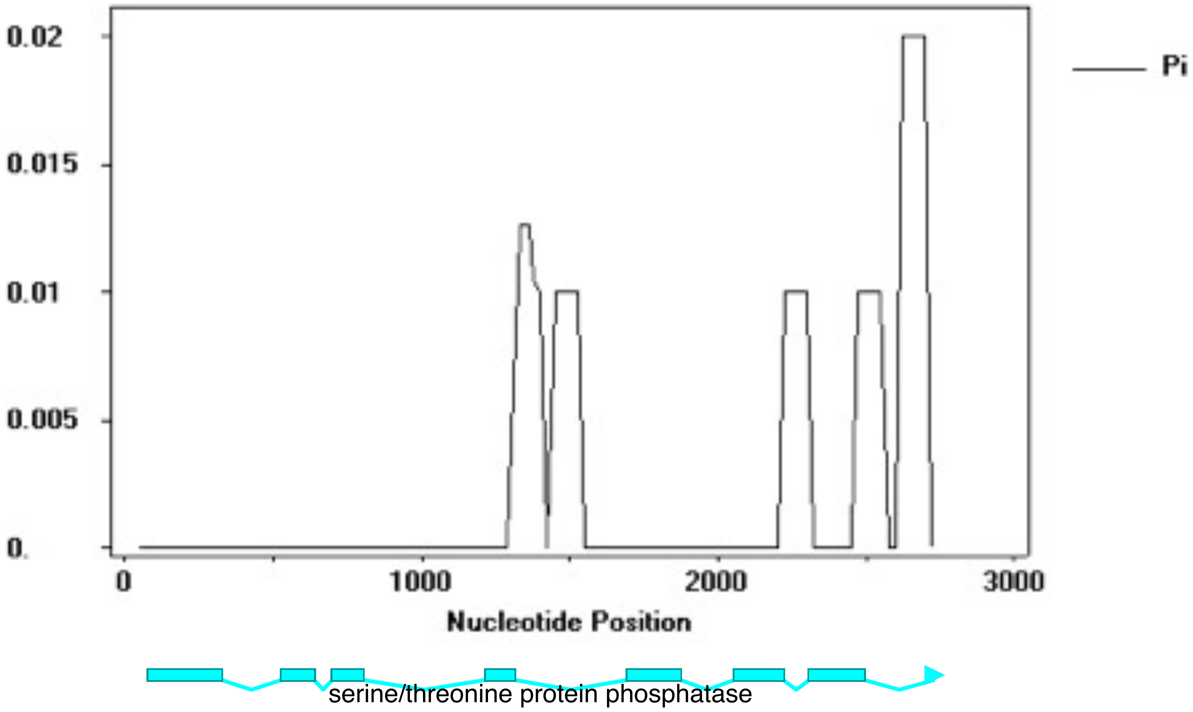
0.02
0.015
0.01
0.005
0.
0
1000
2000
3000
Nucleotide Position
Pi
serine/threonine protein phosphatase

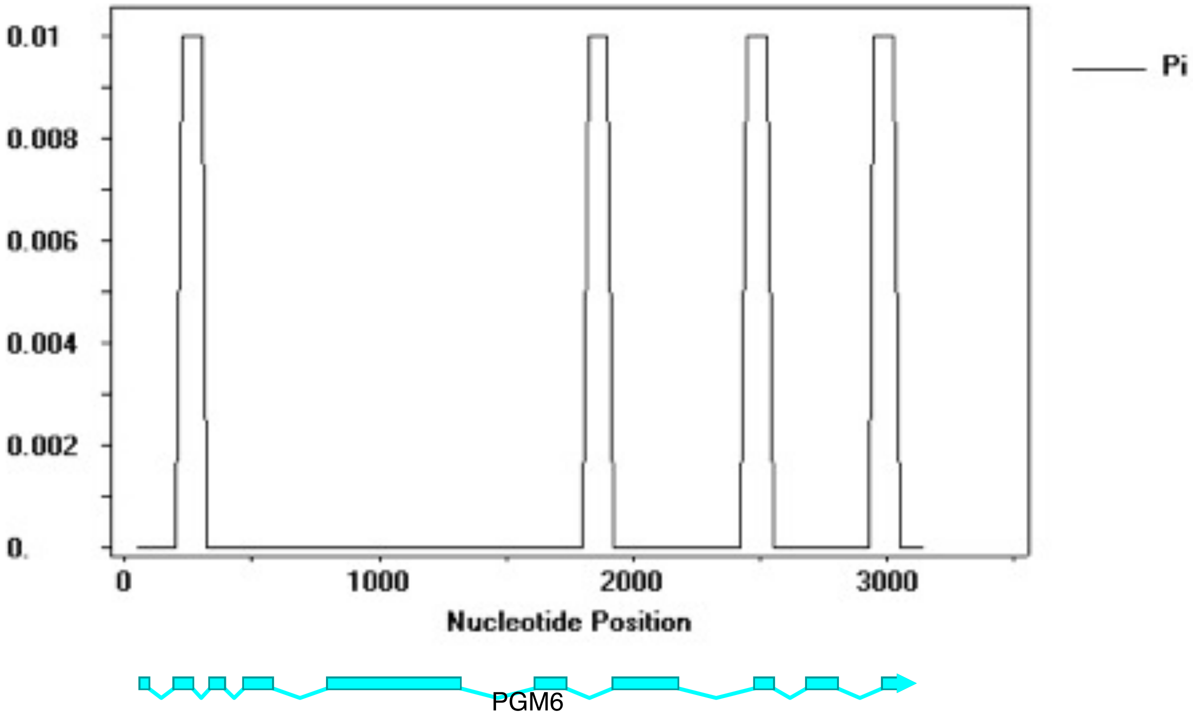
0.01
0.008
0.006
0.004
0.002
0.
0
1000
2000
3000
Nucleotide Position
Pi
PGM6

Figure S4. Sliding window analyses of *Gonium pectorale* gametologs. Pi values in 100 base-window of 25 base-interval were plotted from first to last codons. Each gene model is designated at the bottom of the plot.
