# Supplementary material for: Sequence of the Gonium pectorale Mating Locus Reveals a Complex and Dynamic History of Changes in Volvocine Algal Mating Haplotypes
Source: G3 (Bethesda). 2016 Feb 22;6(5):1179–89. doi: 10.1534/g3.115.026229 (PMC4856071; doi:10.1534/g3.115.026229)
Supplement: Supplemental Material [file supp_g3.115.026229_FigureS5.pdf]

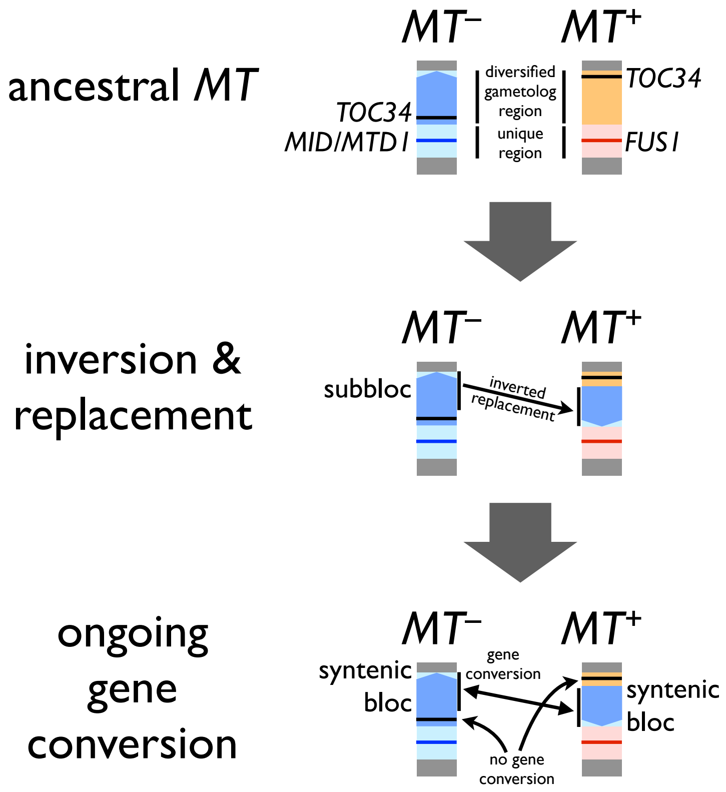

Figure S5. A model for the observed discontinuity in sequence divergence and structure of *G. pectorale MT*. Top panel. The ancestral *MT* had *MID* and *MTD* as *MT*-specific genes and *FUS1* as a *MT*+ specific gene, all within unique regions of each mating type (shaded light blue and pink, respectively). The gametologs (dark blue and orange shaded regions for *MT*- and *MT*+ respectively) had begun to diverge and lose recombination and/or gene conversion. *TOC34* is labeled in black. Middle panel. An inversion and replacement event occurred with a break point between *TOC34* and the adjacent gene, *HSP70B*, that copied *MT*- gametologs into *MT*+. Lower panel. The copied gametolog sequences (dark blue in *MT*+) continue to undergo gene conversion that maintains sequence homogeneity. The *TOC34* locus that was not involved in the copying event remains more diverged due to absent or reduced gene conversion between mating haplotypes (see also **Figure 3B).**
