# Supplementary material for: Sequence of the Gonium pectorale Mating Locus Reveals a Complex and Dynamic History of Changes in Volvocine Algal Mating Haplotypes
Source: G3 (Bethesda). 2016 Feb 22;6(5):1179–89. doi: 10.1534/g3.115.026229 (PMC4856071; doi:10.1534/g3.115.026229)
Supplement: Supplemental Material [file supp_g3.115.026229_FigureS6.pdf]

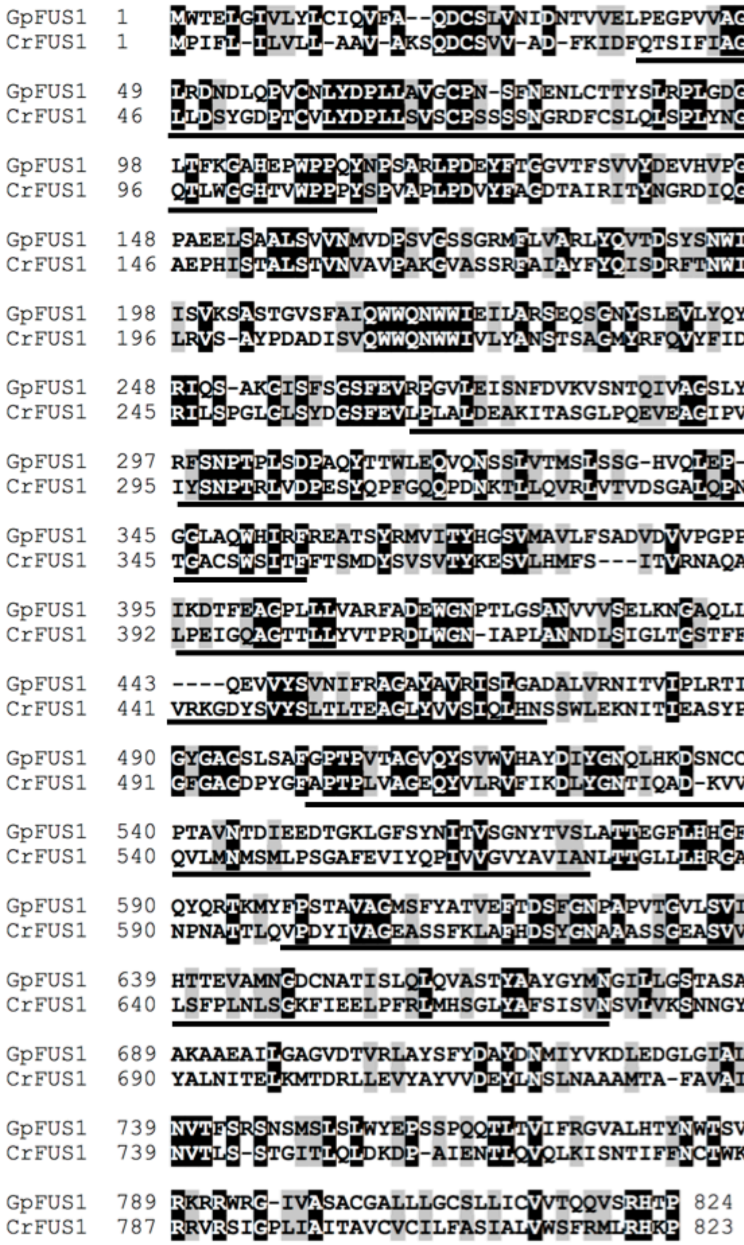

Figure S6. Alignment of the amino acid sequences of the GpFUS1 and CrFUS1 proteins. Identical or similar residues have solid or shaded backgrounds, respectively. Underlined residues are aligned in Figure S7. The GC content of *GpFUS1* CDS and introns are mediate (~50%), showing a common feature found in most of the previously identified mating type-specific genes (*CrFUS1*: 47.7%; *CrMID*: 50.5%; *GpMID*: 52.9%; *GpMTD1*: 55.5%).
