# Supplementary material for: Sequence of the Gonium pectorale Mating Locus Reveals a Complex and Dynamic History of Changes in Volvocine Algal Mating Haplotypes
Source: G3 (Bethesda). 2016 Feb 22;6(5):1179–89. doi: 10.1534/g3.115.026229 (PMC4856071; doi:10.1534/g3.115.026229)
Supplement: Supplemental Material [file supp_g3.115.026229_FigureS7.pdf]

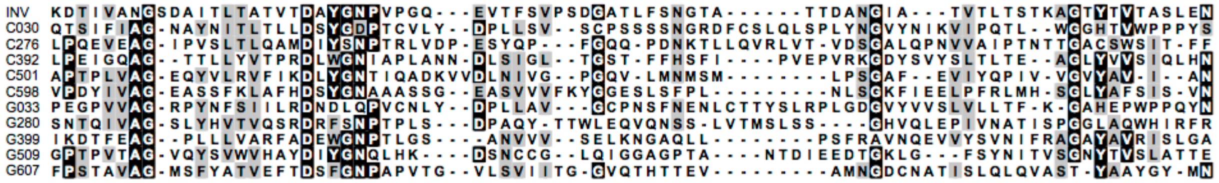

Figure S7. Invasin/intimin immunoglobulin (Ig)-like repeats found in both *Chlamydomonas reinhardtii* and *Gonium pectorale* FUS1 based on Misamore et al. (2003). FUS1 domains and the bacterial invasin/ intimin Ig-like consensus sequence are aligned. "INV" is a consensus sequence based on 25 different, invasin-related BID1, bacterial Ig-like (group 1) domains (accession numbers: 1F00_I, 1CWV_A, gi12513096, gi12516151, gi12519346, gi1706558, gi2125981, gi124714, gi3257750, gi11044949, and gi7462086). The remaining ten sequences, whose positions are indicated by the numbers at the left, are derived from FUS1 (C for CrFUS1; G for GpFUS1). The alignment was manually edited. Identical and similar amino acids are represented by blocked and shaded backgrounds, respectively.
