# Supplementary material for: Sequence of the Gonium pectorale Mating Locus Reveals a Complex and Dynamic History of Changes in Volvocine Algal Mating Haplotypes
Source: G3 (Bethesda). 2016 Feb 22;6(5):1179–89. doi: 10.1534/g3.115.026229 (PMC4856071; doi:10.1534/g3.115.026229)
Supplement: Supplemental Material [file supp_g3.115.026229_FigureS8.pdf]

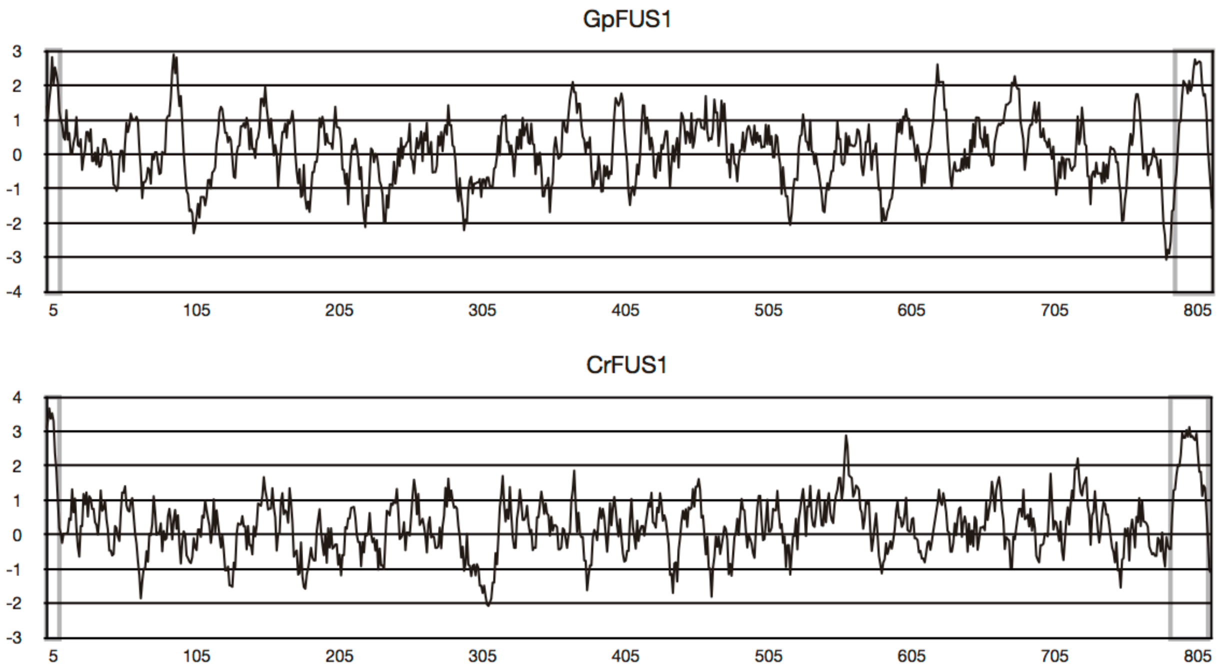

Figure S8. Comparison of FUS1 hydrophobicity. Hydrophobicity plots of GpFUS1 and CrFUS1 by ExPASY are shown. Hydrophobic regions in N- (GpFUS1: 13 residues; CrFUS1: 14 residues) and C-termini (both GpFUS1 and CrFUS1: 29 residues) are boxed in grey. Hydrophobicity plots predict a N-terminal signal peptide and C-terminal transmembrane domain in FUS1 homologs from both species, while secondary structure predictions vary between them.
