# Supplementary figures and images for: Sequence of the Gonium pectorale Mating Locus Reveals a Complex and Dynamic History of Changes in Volvocine Algal Mating Haplotypes
Source: G3 (Bethesda). 2016 Feb 22;6(5):1179–89. doi: 10.1534/g3.115.026229 (PMC4856071; doi:10.1534/g3.115.026229)

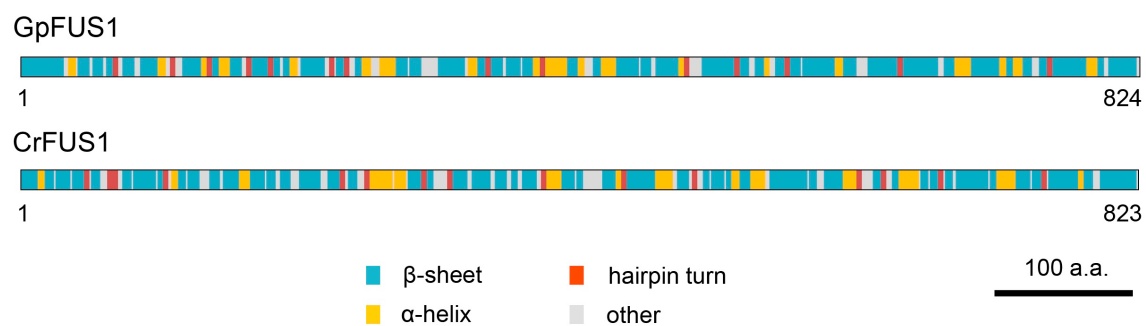

**Figure S9. Schematic comparison of structure predictions for FUS1 proteins.**

Supplement: Supplemental Material [file supp_g3.115.026229_FigureS9.pdf]
