# Supplementary material for: Sequence of the Gonium pectorale Mating Locus Reveals a Complex and Dynamic History of Changes in Volvocine Algal Mating Haplotypes
Source: G3 (Bethesda). 2016 Feb 22;6(5):1179–89. doi: 10.1534/g3.115.026229 (PMC4856071; doi:10.1534/g3.115.026229)
Supplement: Supplemental Material [file supp_g3.115.026229_TableS1.pdf]

Table S1. Primers used in this study. Primers used for AFLP analyses are indicated with their restriction enzymes and digest sizes for Mongolia 1 or 4 genotypes.

Marker or product	Primer name	Oligo DNA sequence	Restriction enzyme for AFLP	Mongolia 1 fragment (approx.)	Mongolia 4 fragment (approx.)
LEU1S	GPLEUFATG	ATGCTGCTCCAAAGGTGGC			
LEU1S	GPLEURTAA	TTACGCCACTTTGATCATGCC			
FUS1	GpFUS1AF	TACCCTGATGGTCGTGCGGAAC			
FUS1	GpFUS1AR	GTTCCGCACGACCATCAGGGTA			
FUS1	GpFUS1BF	AGCGGCAGTTTTGAAGTTCGC			
FUS1	GpFUS1BR	GCGAACTTCAAAACTGCCGCT			
FUS1	GpFUS1CF	AACACCTCTGAGTGATCCCGC			
FUS1	GpFUS1CR	GCGGGATCACTCAGAGGTGTT			
FUS1	YM001F	CACCGTCTACGTGGTGTCCCTT			
FUS1	YM001R	CTATGAGACCCAGCTGTCATGTAG			
PGM6	PGM6-3F	GACCTTCCTGCACATGCT	SmaI	355+185	230+185+125
PGM6	PGM6-4R	CGAGGCCGTCCAATGTC			
SpoVS	SpoVS-1F	CATTGGAGCGGAGAGCATAAA	HindIII	300+200	500
SpoVS	SpoVS-2R	TAGAGCGCCAGACTCTTCTT			
PLC	PLC-1F	GCTGCCATGCTACTTCATCT	ScaI	600	450+150
PLC	PLC-1R	TTGAACGTGTCCAGCTCTATG			
UNC50	UNC50-1F	TCATCGTCGTTTGCTGTACG	NcoI	230+170	400
UNC50	UNC50-2R	CCAAGCAGCAGGAAGTCAA			
PRX1	PRX1-3F	GACCGCTTCAAGGAGTTCAA	EaeI	320	260+60
PRX1	PRX1-4R	TCGACTGCACGTACTGGATA			
MTF1109	MTF1109-1F	CCATGATGAGCGACGAGATTAG	NotI	400	200+200
MTF1109	MTF1109-2R	CCCAGCACCGCAATCTT			
DRG1	MongDRG1F	CAAGAAGCCGCCGAGTATAAC	KpnI	450	290+170
DRG1	MongDRG2R	CGGCGTCCAGGTCTATCTT			
