# Supplementary material for: Sequence of the Gonium pectorale Mating Locus Reveals a Complex and Dynamic History of Changes in Volvocine Algal Mating Haplotypes
Source: G3 (Bethesda). 2016 Feb 22;6(5):1179–89. doi: 10.1534/g3.115.026229 (PMC4856071; doi:10.1534/g3.115.026229)
Supplement: Supplemental Material [file supp_g3.115.026229_TableS2.pdf]

Table S2. Presence or absence of mating type/sex specifically coded genes in volvocine *MT* loci. Blanks (-) indicate absence. *V. carteri MTD1* is considered to be a pseudogene (Ferris et al. 2010).

query/gene name	Volvox	Chlamydomonas	Gonium
FSI1f	Female	-	-
HMG1f	Female	-	-
MTF0821	Female	-	-
MTF0991	Female	-	-
MTF2030	Female	-	-
MTM0097	Male	-	-
MTM0441	Male	-	-
MTM0564	Male	-	-
MTM0665	Male	-	-
MTM0761	Male	-	-
MTM0832	Male	-	-
MTM0897	Male	-	-
MTM0946	Male	-	-
MID	Male	*minus*	*minus*
MTD1	Male (pseudo)	*minus*	*minus*
FUS1	-	*plus*	*plus*
MTA1	-	*plus*	-
EZY2	-	*plus*	-
