# Supplementary material for: Sequence of the Gonium pectorale Mating Locus Reveals a Complex and Dynamic History of Changes in Volvocine Algal Mating Haplotypes
Source: G3 (Bethesda). 2016 Feb 22;6(5):1179–89. doi: 10.1534/g3.115.026229 (PMC4856071; doi:10.1534/g3.115.026229)
Supplement: Supplemental Material [file supp_g3.115.026229_TableS3.pdf]

Table S3. Locations of *Volvox carteri* and *Chlamydomonas reinhardtii MT* R domain genes in *Gonium pectorale*. *V. carteri* gametologs ("query/gene name" and their positions) were used to identify their homologs in *C. reinhardtii* and *G. pectorale*, except for "522875" from *C. reinhardtii* rearranged domain. For *V. carteri*, *MT* and surrounding locations are designated as either linkage group (LG) I rearranged (R), telomere- (T) or centromere- (C) proximal. For *C. reinhardtii*, locations around *MT* are shown in chromosome (Chr.) 6 with C/R/T. *G. pectorale* location are not coordinated to chromosome or linkage groups and represented by scaffold names. Syntenic blocs in *G. pectorale MT* are designated by I, II, III, IV, or v as shown in Figure 2A. *G. pectorale* scaffold00010 is closely linked to *MT*. Genomic locations unlinked to *MT* loci are designated in respective scaffolds. n/d: not detected

query/gene name	*Volvox carteri*	*Chlamydomonas reinhardtii*	*Gonium pectorale*
TOC34	LG I R	Chr. 6 (T)	MT (I)
MAT3	LG I R	Chr. 6 (C)	MT (II)
WDR57	LG I R	Chr. 6 (C)	MT (II)
LEU1S	LG I R	Chr. 6 (R)	MT (II)
ATPvC1	LG I R	Chr. 6 (T)	MT (II)
EIF5Bb	LG I R	Chr. 6 (T)	MT (II)
HSP70b	LG I R	Chr. 6 (T)	MT (II)
METM1	LG I R	Chr. 6 (T)	MT (II)
MME6	LG I R	Chr. 6 (T)	MT (II)
SPS1	LG I R	Chr. 6 (T)	MT (II)
DRG1	LG I R	Chr. 6 (R)	MT (v)
PTC1	LG I R	Chr. 6 (T)	MT (III)
RPL37a	LG I C	Chr. 6 (C)	MT (IV)
ATPvL1	LG I R	Chr. 6 (C)	MT (IV)
FTT2	LG I R	Chr. 6 (C)	MT (IV)
MTF0716	LG I R	Chr. 6 (C)	MT (IV)
MTF1026	LG I R	Chr. 6 (C)	MT (IV)
PGM6	LG I R	Chr. 6 (C)	MT (IV)
SelEF	LG I R	Chr. 6 (C)	MT (IV)
AMPKR1	LG I R	Chr. 6 (T)	MT (IV)
PLC	LG I R	Chr. 6 (C)	scaffold00010
SpoVS	LG I R	Chr. 6 (C)	scaffold00010
VPS53A	LG I R	Chr. 6 (C)	scaffold00010
VPS53B	LG I R	Chr. 6 (C)	scaffold00010
VPS53C	LG I R	Chr. 6 (C)	scaffold00010
VPS53D	LG I R	Chr. 6 (C)	scaffold00010
PAP1	LG I T	Chr. 6 (C)	scaffold00010
AIP1	LG I R	Chr. 6 (C)	scaffold00001
PRX1	LG I R	Chr. 6 (R)	scaffold00001
UNC50	LG I R	Chr. 6 (T)	scaffold00002
RGP1	LG I R	Chr. 6 (C)	scaffold00003
MTF1320	LG I R	Chr. 6 (C)	scaffold00003
MTF1109	LG I R	Chr. 6 (T)	scaffold00003
MTF0870	LG I R	n/d	scaffold00004
BBS2	LG I C	Chr. 6 (C)	scaffold00005
ARP4	LG I R	Chr. 6 (T)	scaffold00005
MTF1484	LG I R	Chr. 6 (T)	scaffold00005
NMDA1	LG I C	Chr. 6 (R)	scaffold00006
PR46a	LG I C	Chr. 6 (R)	scaffold00007
PDK1	scaffold_11	Chr. 6 (R)	scaffold00007
ALB3_1	LG I R	Chr. 6 (T)	scaffold00007
MTF0992	LG I R	n/d	scaffold00007
GCSH	LG I C	Chr. 6 (R)	scaffold00008
CGL70	scaffold_47	Chr. 6 (R)	scaffold00009
PR46b	LG I C	Chr. 6 (R)	scaffold00014
UTP1	LG I R	Chr. 6 (R)	scaffold00015
LPS1	scaffold_20	Chr. 6 (R)	scaffold00021
MOT41	LG I R	Chr. 6 (T)	scaffold00022
SPL2	LG I R	Chr. 6 (R)	scaffold00026
MTF0674	LG I R	n/d	scaffold00027
BFR1	LG I R	Chr. 6 (C)	scaffold00029
THI10	LG I R	Chr. 6 (C)	scaffold00029
MTF1436	LG I R	Chr. 6 (T)	scaffold00033
FA1	LG I R	Chr. 6 (C)	scaffold00043
PRP4	LG I T	Chr. 6 (T)	scaffold00045
NMT1	LG I R	Chr. 6 (T)	scaffold00046
DHC1b	LG I R	Chr. 6 (T)	scaffold00049
L7Ae	LG I R	Chr. 6 (T)	scaffold00053
CRB1	LG I R	Chr. 6 (T)	scaffold00055
UBCH1	LG I R	Chr. 6 (R)	scaffold00064
EFG8	LG I R	Chr. 6 (C)	scaffold00071
MAPKK1	LG I R	Chr. 6 (T)	scaffold00090
FAP75	LG I T	Chr. 6 (T)	scaffold00090
CGL55	LG I R	Chr. 6 (T)	scaffold00091
OTU2a	scaffold_1	Chr. 6 (R)	scaffold00113
PIP5K1	LG I T	Chr. 6 (T)	scaffold00116
MTF1458	LG I R	Chr. 6 (C)	scaffold00191
SAD1	LG I R	Chr. 6 (C)	scaffold00324
522872	scaffold_8	Chr. 6 (R)	scaffold00373
RPS3A2	LG I T	Chr. 13	scaffold00462
PSF2	LG I R	Chr. 6 (T)	scaffold00652
MADS2	LG I C	Chr. 6 (R)	scaffold00785
DLA3	n/d	Chr. 6 (R)	scaffold00802
MTF1733	LG I R	Chr. 6 (T)	scaffold01026
PKY1	LG I C	Chr. 6 (R)	scaffold01360
HPRG1	LG I R	Chr. 6 (R)	scaffold01894
UCP2	LG I R	Chr. 6 (C)	n/d
522875	n/d	Chr. 6 (R)	n/d
MTF0684	LG I R	Chr. 6 (T)	n/d
MTF0822	LG I R	Chr. 6 (T)	n/d
MT0044	LG I T	Chr. 6 (T)	n/d
MT0045	LG I T	Chr. 6 (T)	n/d
MT2177	LG I C	n/d	n/d
MT2215	LG I C	n/d	n/d
MTF0709	LG I R	n/d	n/d
MT0083	LG I T	n/d	n/d
