# Supplementary material for: Sequence of the Gonium pectorale Mating Locus Reveals a Complex and Dynamic History of Changes in Volvocine Algal Mating Haplotypes
Source: G3 (Bethesda). 2016 Feb 22;6(5):1179–89. doi: 10.1534/g3.115.026229 (PMC4856071; doi:10.1534/g3.115.026229)
Supplement: Supplemental Material [file supp_g3.115.026229_TableS4.pdf]

Table S4. Non-synonymous (dN) and synonymous (dS) substitutions of gametologs in *Gonium pectorale*.

Name	dN	SE	dS	SE
AMPKR1	0.0004	0.0003	0.0008	0.0008
ATPvC1	0	0	0.004	0.004
ATPvL1	0	0	0.0068	0.0068
DRG1	0	0	0.0048	0.0049
EIF5Bb	0.0009	0.0009	0	0
FTT2	0	0	0	0
GMTp03	0.0012	0.0012	0	0
GMTp14	0.0007	0.0005	0.0018	0.0018
GMTp19	0	0	0	0
HSP70B *	0.0006	0.0006	0.0678	0.0151
LEU1S	0	0	0.02	0.0116
MAT3	0.0005	0.0005	0	0
METM1	0	0	0.0109	0.0077
MME6	0.0013	0.0009	0.0118	0.0059
PGM6	0	0	0	0
PTC1	0	0	0.0068	0.0034
RPL37A	0	0	0	0
SelEF	0.0007	0.0007	0	0
SPS1	0	0	0	0
TOC34 *	0.0623	0.0097	0.5549	0.0785
WDR57	0.0014	0.0014	0.0044	0.0044
